# Supplementary material for: Context-specific eQTLs provide deeper insight into causal genes underlying shared genetic architecture of COVID-19 and idiopathic pulmonary fibrosis
Source: HGG Adv. 2025 Jan 27;6(2):100410. doi: 10.1016/j.xhgg.2025.100410 (PMC11872446; doi:10.1016/j.xhgg.2025.100410)
Supplement: Document S1. Figures S1–S3 [file mmc1.pdf]

HGGA, Volume *6*

Supplemental information

Context-specific eQTLs provide deeper insight into causal genes underlying shared genetic architecture of COVID-19 and idiopathic pulmonary fibrosis

Trisha Dalapati, Liuyang Wang, Angela G. Jones, Jonathan Cardwell, Iain R. Konigsberg, Yohan Bossé, Don D. Sin, Wim Timens, Ke Hao, Ivana Yang, and Dennis C. Ko

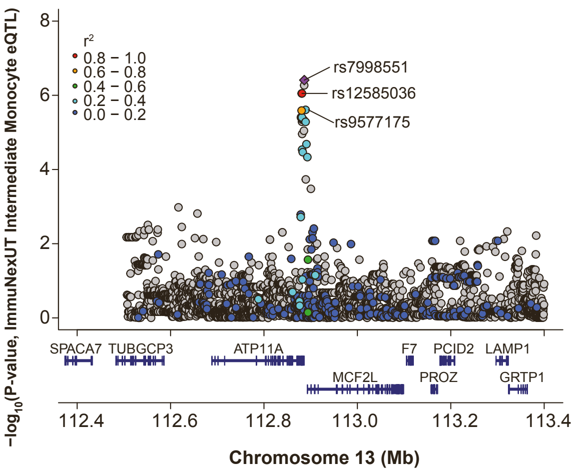

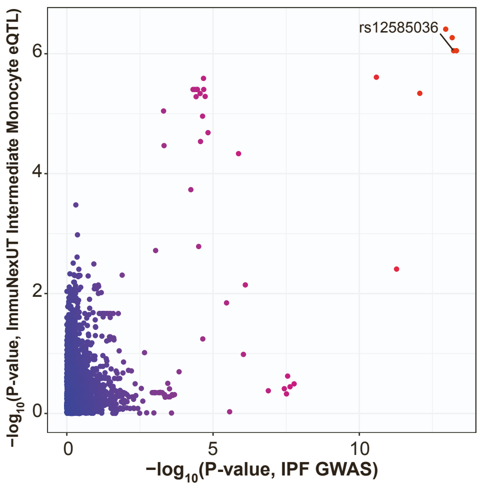

C

COLOC of the ImmuNexUT Intermediate Monocyte eQTL and Disease GWAS Signals at *ATP11A*

rsID	Disease	PP3	PP4	PP4/PP3	Interpretation
**rs12585036**	IPF	9.83×10^{-3}	0.988	1.01×10^{2}	Traits share a single causal variant.
	COVID-19	8.88×10^{-3}	0.989	1.11×10^{2}	Traits share a single causal variant.

Figure S1: Colocalization at rs12585036 reveals *ATP11A* as a causal gene in monocytes from a Japanese cohort.

(A) rs12585036 is a top eQTL and in strong LD with the lead eQTL (rs7998551) for *ATP11A* in monocytes, most significantly in intermediate monocytes, in the ImmuNexUT eQTL atlas.

(B) Comparison of $-\log_{10}$(p-values) from the IPF GWAS and intermediate monocytic *ATP11A*-eQTLs shows rs12585036 as a top shared SNP in ImmuNexUT.

(C) COLOC indicates strong colocalization between the disease GWAS and monocytic *ATP11A*-eQTLs signals in ImmuNexUT with PP4 > 0.900 and PP4/PP3 > 5.00

P-values are $-\log_{10}$ transformed. Linkage disequilibrium information for the Japanese population was obtained from LDlink and is relative to the lead variant in each plot.

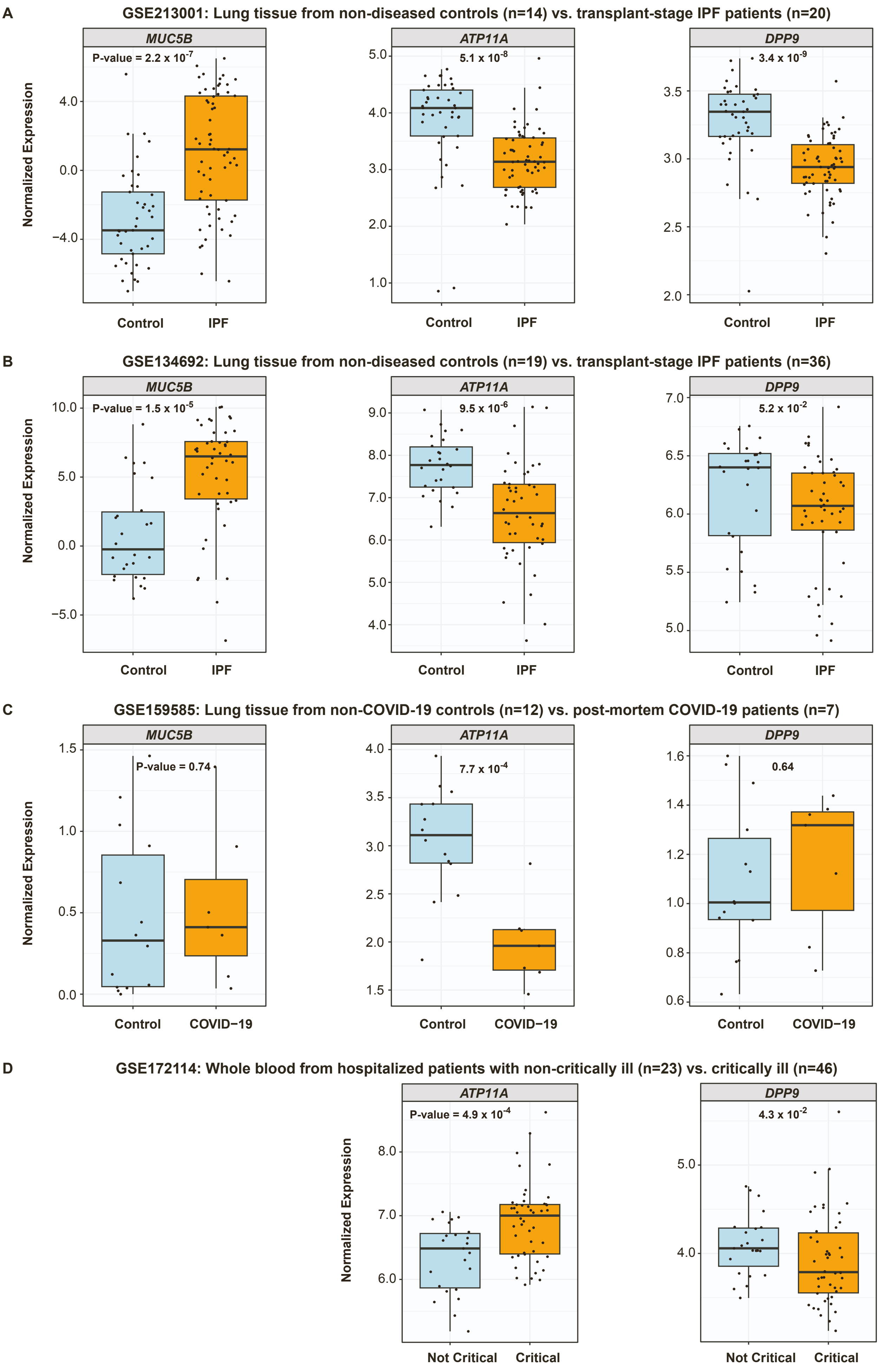
A
GSE213001: Lung tissue from non-diseased controls (n=14) vs. transplant-stage IPF patients (n=20)
MUC5B
P-value = 2.2 x 10-7
ATP11A
5.1 x 10-8
DPP9
3.4 x 10-9
Normalized Expression
Control
IPF
B
GSE134692: Lung tissue from non-diseased controls (n=19) vs. transplant-stage IPF patients (n=36)
MUC5B
P-value = 1.5 x 10-5
ATP11A
9.5 x 10-6
DPP9
5.2 x 10-2
Normalized Expression
Control
IPF
C
GSE159585: Lung tissue from non-COVID-19 controls (n=12) vs. post-mortem COVID-19 patients (n=7)
MUC5B
P-value = 0.74
ATP11A
7.7 x 10-4
DPP9
0.64
Normalized Expression
Control
COVID−19
D
GSE172114: Whole blood from hospitalized patients with non-critically ill (n=23) vs. critically ill (n=46)
ATP11A
P-value = 4.9 x 10-4
DPP9
4.3 x 10-2
Normalized Expression
Not Critical
Critical

Figure S2: Assessment of causal genes in bulk control and diseased tissue from independent studies.

Boxplots depict normalized expression of causal genes by diagnosis from:

(A) GSE213001, which included lung tissue from non-diseased controls (n=14) and IPF patients undergoing lung transplant (n=20).

(B) GSE134692, which included lung tissue from non-diseased controls (n=19) and IPF patients undergoing lung transplant (n=36).

(C) GSE159585, which included lung tissue from non-diseased controls (n=12) and from patients who died from COVID-19 (n=7).

(D) GSE172114, which included whole blood samples from hospitalized patients with non-critically ill COVID-19 (n=23) and patients in the intensive care unit with critically ill COVID-19 (n=46).

P-values were calculated using the Mann-Whitney U test.

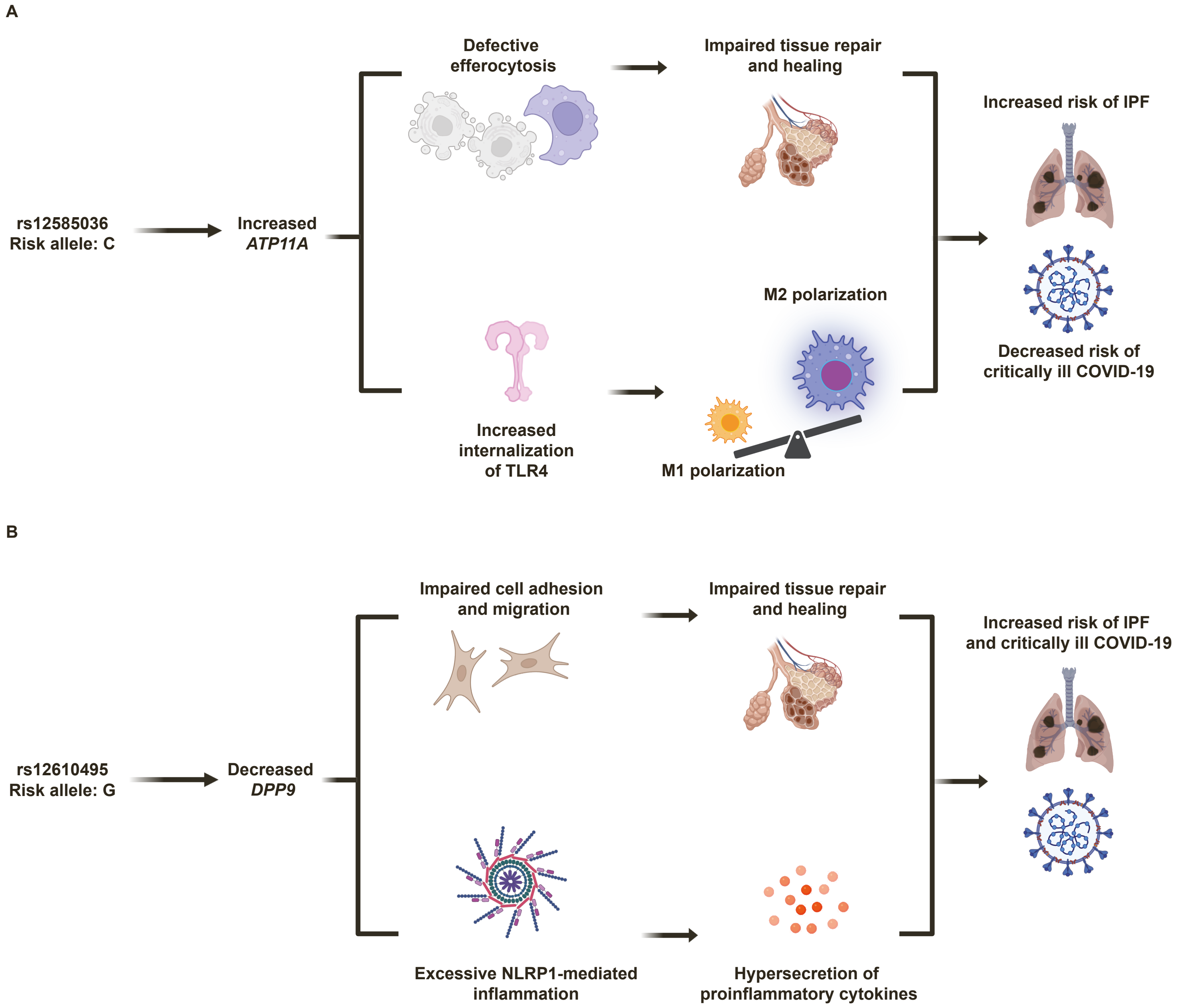

A
rs12585036
Risk allele: C
Increased
ATP11A
Defective
efferocytosis
Impaired tissue repair
and healing
Increased
internalization
of TLR4
M2 polarization
M1 polarization
Increased risk of IPF
Decreased risk of
critically ill COVID-19
B
rs12610495
Risk allele: G
Decreased
DPP9
Impaired cell adhesion
and migration
Impaired tissue repair
and healing
Excessive NLRP1-mediated
inflammation
Hypersecretion of
proinflammatory cytokines
Increased risk of IPF
and critically ill COVID-19

Figure S3: Hypothesized mechanisms linking disease-associated SNPs to causal genes, *ATP11A* and *DPP9*.

(A) The C allele of rs12585036 is associated with increased *ATP11A*, leading to 1) defective efferocytosis and impaired lung tissue repair and 2) increased internalization of toll-like receptor 4 and increased M2-macrophage polarization. These mechanisms lead to increased risk of IPF and protection from critically ill COVID-19.

(B) The G allele of rs12610495 is associated with decreased *DPP9*, leading to 1) impaired cell adhesion and migration affecting lung tissue repair, and 2) excessive NLRP1-inflammasome activity and proinflammatory cytokine production. These mechanisms lead to increased risk of both IPF and critically ill COVID-19.
