# Supplementary material for: Context-specific eQTLs provide deeper insight into causal genes underlying shared genetic architecture of COVID-19 and idiopathic pulmonary fibrosis
Source: HGG Adv. 2025 Jan 27;6(2):100410. doi: 10.1016/j.xhgg.2025.100410 (PMC11872446; doi:10.1016/j.xhgg.2025.100410)
Supplement: Document S2. Article plus supplemental information [file mmc3.pdf]

HGG Advances

ARTICLE

Context-specific eQTLs provide deeper insight into causal genes underlying shared genetic architecture of COVID-19 and idiopathic pulmonary fibrosis

Trisha Dalapati,[1,2] Liuyang Wang,[2] Angela G. Jones,[2,3] Jonathan Cardwell,[4] Iain R. Konigsberg,[4] Yohan Bossé,[5] Don D. Sin,[6] Wim Timens,[7] Ke Hao,[8] Ivana Yang,[4] and Dennis C. Ko[2,3,9,10,*]

Summary

Most genetic variants identified through genome-wide association studies (GWASs) are suspected to be regulatory in nature, but only a small fraction colocalize with expression quantitative trait loci (eQTLs, variants associated with expression of a gene). Therefore, it is hypothesized but largely untested that integration of disease GWAS with context-specific eQTLs will reveal the underlying genes driving disease associations. We used colocalization and transcriptomic analyses to identify shared genetic variants and likely causal genes associated with critically ill COVID-19 and idiopathic pulmonary fibrosis. We first identified five genome-wide significant variants associated with both diseases. Four of the variants did not demonstrate clear colocalization between GWAS and healthy lung eQTL signals. Instead, two of the four variants colocalized only in cell type- and disease-specific eQTL datasets. These analyses pointed to higher *ATP11A* expression from the C allele of rs12585036, in monocytes and in lung tissue from primarily smokers, which increased risk of idiopathic pulmonary fibrosis (IPF) and decreased risk of critically ill COVID-19. We also found lower *DPP9* expression (and higher methylation at a specific CpG) from the G allele of rs12610495, acting in fibroblasts and in IPF lungs, and increased risk of IPF and critically ill COVID-19. We further found differential expression of the identified causal genes in diseased lungs when compared to non-diseased lungs, specifically in epithelial and immune cell types. These findings highlight the power of integrating GWASs, context-specific eQTLs, and transcriptomics of diseased tissue to harness human genetic variation to identify causal genes and where they function during multiple diseases.

Introduction

Genome-wide association studies (GWASs) are a powerful approach to identify relationships between disease phenotypes and genetic variants, most commonly single nucleotide polymorphisms (SNPs). Elucidating associated SNPs can reveal unanticipated underlying pathophysiology of disease, unbiased by preconceived notions of plausible biology, and may lead to informed drug targets and therapies. Indeed, success along the drug development pipeline increases with human genetic evidence.[1] In 2021, two-thirds of the Food and Drug Administration-approved drugs were supported by human genetic evidence.[2] Beyond associations with individual diseases, multiple independent GWASs can be integrated to identify pleiotropy—the phenomenon where a single genetic locus affects multiple diseases or traits that are otherwise considered unrelated.[3,4] Pleiotropy can reveal shared pathogenic mechanisms among different diseases. Understanding these mechanisms and identifying potential therapeutic targets require the identification of the causal genes associated with the genetic variant.

One method to identify putative casual genes is to measure the degree of colocalization between disease GWASs and molecular quantitative trait loci (QTLs), such as expression QTLs (eQTLs), using statistical frameworks that integrate summary statistics to determine if overlapping signals are due to the same causal SNP.[5–7] While the chain of causality implied by colocalization makes intuitive sense (an SNP affects transcription of a gene to impact disease), there is surprisingly poor colocalization between disease GWASs and eQTL signals.[8] One possible reason for lack of colocalization includes using eQTL datasets from unsuitable contexts. For example, cell- and disease-specific eQTLs may be required.[8] Alternatively, other kinds of molecular QTLs, such as methylation, splicing, or protein QTLs (mQTLs, sQTLs, pQTLs, respectively), may be more causally important in certain disease pathogenesis.[8,9]

Here, we focused on pleiotropic SNPs associated with both critically ill COVID-19 and idiopathic pulmonary fibrosis (IPF) and formally tested colocalization with context-specific eQTLs to identify the likely causal genes. Critically ill COVID-19 was defined as individuals requiring respiratory support in the hospital or those

[1]Medical Scientist Training Program, Duke University School of Medicine, Durham, NC, USA; [2]Department of Molecular Genetics and Microbiology, Duke University School of Medicine, Durham, NC, USA; [3]University Program in Genetics and Genomics, Duke University, Durham, NC, USA; [4]Department of Biomedical Informatics, School of Medicine, University of Colorado Anschutz Medical Campus, Aurora, CO, USA; [5]Institut universitaire de cardiologie et de pneumologie de Québec – Université Laval, Department of Molecular Medicine, Québec City, QC, Canada; [6]Center for Heart Lung Innovation, University of British Columbia and St. Paul's Hospital, Vancouver, BC, Canada; [7]Department of Pathology and Medical Biology, University Medical Center Groningen, University of Groningen, Groningen, the Netherlands; [8]Department of Genetics and Genomic Sciences, Icahn School of Medicine at Mount Sinai, New York, NY, United States; [9]Division of Infectious Diseases, Department of Medicine, Duke University School of Medicine, Durham, NC, USA
[10]Lead contact
*Correspondence: dennis.ko@duke.edu
https://doi.org/10.1016/j.xhgg.2025.100410.
© 2025 The Author(s). Published by Elsevier Inc. on behalf of American Society of Human Genetics.
This is an open access article under the CC BY license (http://creativecommons.org/licenses/by/4.0/).

who died from disease by the COVID-19 Host Genetics Initiative (HGI).[10] We previously developed a software called the interactive Cross-Phenotype Analysis of GWAS database (iCPAGdb) to study pleiotropy by comprehensively identifying shared association signals between user-uploaded GWAS and all publicly cataloged GWAS summary statistics.[11,12] Using the first COVID-19 GWAS published by the Severe Covid-19 GWAS Group[13] and the highest-powered IPF GWAS at the time,[14] we identified a shared signal in *DPP9*, rs12610495, associated at a suggestive threshold with severe COVID-19 (p value $= 5.20 \times 10^{-6}$)[13] and at genome-wide significance with IPF (p value $= 2.92 \times 10^{-12}$).[12,14] The *DPP9* locus was later confirmed to be associated with critically ill COVID-19 at genome-wide significance.[15–17] Others have also confirmed and expanded on the shared genetic associations between critically ill COVID-19 and IPF.[12,18–20]

Notably, post-COVID-19 pulmonary fibrosis (PCPF) is clinically reminiscent of IPF.[21] PCPF, which occurs due to irreversible lung scarring and stiffening, causes progressive difficulties in breathing and ultimately necessitates lung transplantation.[21,22] Critical illness is a major risk factor for PCPF.[23] A meta-analysis of observational studies of pulmonary fibrosis among recovering hospitalized COVID-19 patients, including individuals who required respiratory support, reported that nearly 45% showed evidence of long-term respiratory symptoms and imaging findings of fibrosis.[23] In fact, drugs for IPF, including pirfenidone and nintedanib, have been coopted during emergency treatment of PCPF.[24] Thus, shared genetic associations between critically ill COVID-19 and IPF might reflect common pathophysiology that could be utilized for therapeutic benefit in both diseases.

In this study, we leveraged iCPAGdb again with the most current, highest-powered COVID-19 GWAS (COVID-19 HGI release 7)[10] and IPF *meta*-GWAS[25] to determine whether additional loci are associated with both diseases. We identified five shared loci, including the *DPP9* locus containing rs12610495, that are likely due to the same causal variants based on colocalization analysis. For two of the variants, their risk alleles are reversed in COVID-19 and IPF, highlighting that a shared signal may have different functions in the two diseases and that the pulmonary damage from COVID-19 is likely distinct from IPF. We next systematically identified the likely causal genes underlying the shared genetic architecture between critically ill COVID-19 and IPF by performing colocalization analysis using bulk, single-cell, and disease-specific eQTL datasets. For two genes, *ATP11A* and *DPP9,* we found colocalization of GWAS and eQTL signals only in a cell type- and disease-specific context. Therefore, context-specific eQTLs are critical for identifying the causal genes underlying the shared genetic architecture of critically ill COVID-19 and IPF and may lead to new insights connecting the diseases.

Material and methods

GWAS summary statistics and QTL datasets

All datasets used in this study and accession information are summarized in Table S1. The genome coordinates in all datasets were lifted to GRCh38 using the R packages "liftOver"[26] and "rtracklayer."[27] Of note, the most recent GWAS summary statistics from the COVID-19 HGI release 7 included controls from the 23andMe research cohort, making it the most well-powered COVID-19 GWAS to date. However, only the top 10,000 SNPs were released in the summary statistics including the 23andMe subjects. For analyses using GTEx v8, we obtained the "Tissue-Specific All SNP Gene Associations" files via the Google Cloud Platform, which included nonsignificant associations not searchable on the web browser.[28]

In addition to the IPF eQTL and mQTL summary statistics, Borie et al. provided *DPP9* expression and cg07317664 methylation data.[29] p values from eQTL and mQTL summary statistics were generated using FastQTL analysis with the top 30 Probabilistic Estimation of Expression Residuals (PEER) factors, age, sex, and the top four genotypic principal components regressed out of the omics data prior to the analysis. However, the values plotted for gene expression and methylation did not have the effects of the PEER factors regressed out. For eQTL data from Borie et al.,[29] normalized RNA TPM values were plotted (transcripts per million after trimmed mean of M values [TMM] normalization across samples and inverse normal transformation on a per-gene basis). For mQTL data from Borie et al.,[29] normalized methylation beta referred to beta values of DNA methylation level measured on Illumina arrays (scale 0–1) after SeSame[30] data preprocessing and normalization.

Discovery of pleiotropic SNPs using iCPAGdb

We previously reported the development of iCPAGdb which identified cross-phenotypic associations across 4,400 traits loaded from the NHGRI-EBI GWAS Catalog.[11,12] For this study, critically ill COVID-19 summary statistics were formatted for the web browser (http://cpag.oit.duke.edu/explore/app/) and uploaded as the User-Supplied GWAS for "GWAS source one." NHGRI was selected for "GWAS source two." P value thresholds (denoted on the iCPAGdb website as "P-threshold$_1$ (factor$_1$ $\times$ 10^{-x1})" for "GWAS source one" and "P-threshold$_2$ (factor$_2$ X 10^{-x2})" for "GWAS source two") were set to genome-wide significant (5×10^{-8}; 5 for "factor$_1$" and "factor$_2$" and 8 for "^{x1}" and "^{x2}"). "European" was chosen for "LD 1000 Genomes population" as the linkage disequilibrium (LD) population since the IPF GWAS was based on European ancestry. Additionally, the command line version of iCPAGdb (https://github.com/tbalmat/iCPAGdb) was utilized to directly compare the critically ill COVID-19 and IPF *meta*-GWAS, which became publicly available after the publication of iCPAGdb.

The full results from iCPAGdb after uploading the critically ill COVID-19 summary statistics are listed in Table S2. The fold enrichment for each trait pair was calculated by dividing the number of shared variants ("Nshare_all"; column G) by the number of expected shared variants ("N_expected"; column H). The number of expected shared variants in iCPAGdb is calculated by dividing the product of the number of independent SNPs associated with trait 1 ("n1_pcut"; column C) and trait 2 ("n2_pcut"; column D) by the effective number of independent SNPs associated with the user-selected LD population (European, African, or Asian).

The number of independent SNPs associated with each population was obtained from Table 4 (1000 Genomes, M_e) from Li et al.[31]

Identification of candidate eGenes and LD proxies

The five SNPs shared between COVID-19 and IPF identified by iCPAGdb were queried in the eQTL datasets in Table S1. We checked whether each SNP was a conditionally independent eQTL in each of the 54 tissues in GTEx v8. Next, using the GTEx web browser, we downloaded all eGenes associated with each SNP. We then searched for conditionally independent eQTLs across the 54 tissues associated with the list of eGenes. We used LDmatrix to check if any of the conditionally independent eQTLs were in LD with our SNPs and could serve as LD proxies ($r^2 > 0.50$) (Table S3). For rs12585036 and rs12610495, the only conditionally independent SNPs in LD were eQTLs for *ATP11A* and *DPP9*, respectively. Two conditionally independent SNPs were in perfect LD with rs2897075 and were eQTLs for *ZKSCAN1* and *COPS6*. rs1105569 was in strong LD ($r^2 > 0.90$) with 330 conditionally independent eQTLs associated with 23 eGenes across 49 tissues, underscoring the challenges in identifying causal genes in the 17q21.31 inversion supergene.[32] For this locus, we further filtered conditionally independent eQTL in strong LD in tissues of interest for COVID-19 and IPF pathogenesis: fibroblasts, lung, lymphocytes, and whole blood. From literature review, we identified one additional eGene, *TRIM4*, associated with rs2897075[18] and two additional eGenes, *CRHR1* and *SPPL2C*, associated with rs1105569.[32] Altogether, we focused on 15 candidate protein-coding eGenes for our five SNPs of interest (Table S4).

Colocalization analysis

We applied the colocalization analysis (COLOC) of Giambartolomei et al., using the R package "coloc,"[6] to determine if the associations identified by iCPAGdb were due to the same causal SNP. COLOC uses a Bayesian framework to calculate the posterior probabilities that two traits are not associated in the locus of interest (PP0), only one trait is associated in the locus (PP1 and PP2), both traits are associated at the locus but with different, independent causal variants (PP3), or both traits are associated with a single causal variant in the locus (PP4). For COLOC using only GWAS summary statistics, we filtered SNPs within a 1 megabase (Mb) window from the SNP of interest. For COLOC using eQTL datasets, we filtered all eQTLs for a candidate eGene and then filtered SNPs within a 1-Mb window from the SNP of interest. We ran the COLOC "coloc.abf" function using the default prior parameters, $p1 = 1 \times 10^{-4}$, $p2 = 1 \times 10^{-4}$, and $p12 = 1 \times 10^{-5}$ for all analyses. PP4 between 0.300 and 0.700 was interpreted as "limited evidence of colocalization"; 0.700 to 0.900 was interpreted as "likely to share a single causal variant"; and PP4 > 0.900 was interpreted as "sharing a single causal variant." The PP4/PP3 measured the intensity of the colocalization signal with values ≥ 5.00 indicating further support for colocalization and ≥ 3.00 suggesting likely colocalization.[9,33]

IPF and COVID-19 transcriptomic analysis

We utilized publicly available bulk RNA datasets from lung or blood from patients diagnosed with COVID-19 and IPF. Normalized counts were downloaded from GEO (GEO: GSE213001, GEO: GSE134692, GEO: GSE172114).[34–36] When normalized counts were unavailable, raw counts were downloaded (GEO: GSE159585).[37] Genes with counts less than five were filtered out, and the remaining gene counts were normalized using DESeq2.[38] We utilized publicly available single-cell (sc) and single-nucleus (sn) RNA-sequencing (RNA-seq) datasets to investigate causal gene expression in IPF and COVID-19 lungs compared with healthy controls. An scRNA-seq dataset for IPF from Adams et al.[39] containing 243,472 cells from 32 IPF lungs and 28 control donor lungs was downloaded from GEO (GEO: GSE136831). A snRNA-seq dataset for lethal COVID-19 from Melms et al.[40] containing 116,314 nuclei from 19 lethal COVID-19 lungs and seven control lungs was downloaded from the Broad Institute Single Cell Portal.[41] Previously annotated cell-type clusters were used to create expression dot plots and calculate cell-type proportions within each donor with the Python package "scanpy."[42] Only cell-type clusters with at least 10 cells and cells with at least 500 total counts were retained for downstream differential expression analyses. We then pseudobulked gene counts by donor and cell type using the Python package "decoupleR."[43] Differential expression analysis between diseased and healthy lungs was conducted in the Python package "pyDESeq2"[44] that implements the traditional DESeq2[38] workflow. Cell-type proportions calculated from the datasets are in Tables S5 and S6. Differential expression results are in Tables S7 and S8.

Data visualization

All visualizations were created in R version 4.3.0. Lollipop plots, boxplots, and $-\log_{10}(p$ value) scatterplots were generated using "ggplot2."[45] Manhattan plots and LocusZoom plots were created using "fastman"[46] and "locuszoomr," respectively.

Results

Identification of loci shared between critically ill COVID-19 and idiopathic pulmonary fibrosis

The underlying causal genes and mechanisms for the shared loci associated with critically ill COVID-19 and IPF have not been systematically identified. The COVID-19 HGI integrated 82 studies from 35 countries to create the largest cohort to date for risk, hospitalization, and critical illness.[10,47,48] Herein, we studied the critically ill COVID-19 trait (cases = 18,152, controls = 1,145,546) as severe disease is a risk factor for developing lung fibrosis. iCPAGdb revealed shared genetic associations between critically ill COVID-19 and multiple human diseases (Figure 1A; Table S2). Most interestingly, critically ill COVID-19 significantly overlapped with several pulmonary traits (Figure 1B), including interstitial lung disease (ILD) and IPF at five loci "(ILD: p value $= 6.05 \times 10^{-15}$ after Benjamini-Hochberg Procedure; 3,774.0-fold enrichment; IPF: p value $= 1.56 \times 10^{-14}$ after Benjamini-Hochberg Procedure; 4,780.0-fold enrichment) (Figures 1C and 1D). Importantly, the risk allele was reversed for two of the five SNPs, rs35705950 and rs12585036. The odds ratio at each shared locus was also generally larger for IPF, highlighting the complex disease mechanisms linking COVID-19 and IPF (Figure 1C).

Although regional association plots visually suggested colocalizing signals in critically ill COVID-19 and IPF (Figures 2A–2E), shared associations cannot be assumed to be driven by the same causal SNP. Overlapping GWAS

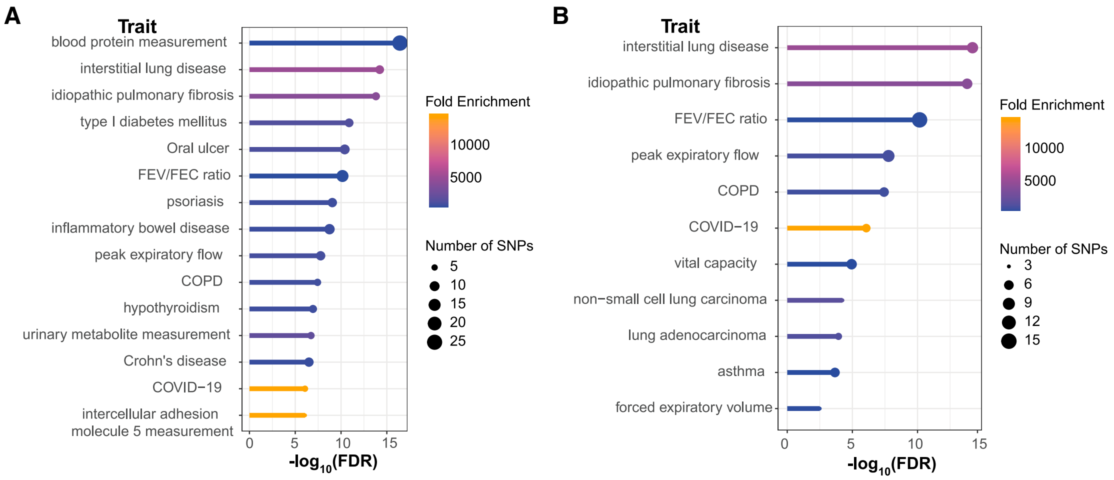

C

Loci shared between IPF and Critically Ill COVID-19							
rsID	Chr: position (GRCh38)	P-value IPF GWAS	Risk Allele - IPF OR	P-value COVID GWAS	Risk Allele - COVID OR	GENCODE Track Location	Functional Annotation
**rs35705950**	11:1219991:G:T	9.0×10^{-418}	T - 5.1	3.6×10^{-14}	G - 1.2	*MUC5B*	
**rs12585036**	13:112881427:C:T	6.0×10^{-14}	C - 1.3	9.3×10^{-19}	T - 1.2	*ATP11A*	Intronic
**rs12610495**	19:4717660:A:G	2.6×10^{-16}	G - 1.3	5.5×10^{-59}	G - 1.3	*DPP9*	Intronic
**rs2897075**	7:100032719:C:T	1.8×10^{-21}	T - 1.3	3.8×10^{-10}	T - 1.1	*ZKSCAN1*	Intronic
**rs1105569**	17:45716022:C:T	4.5×10^{-19}	C - 1.4	1.0×10^{-14}	C - 1.1	*LINC02210-CRHR1*	Intronic

D

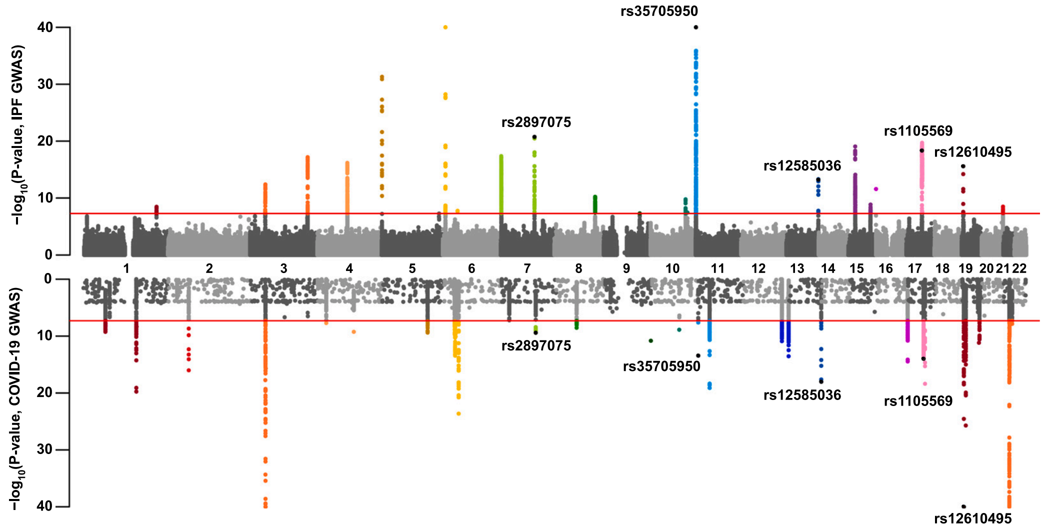

Figure 1. iCPAGdb reveals associations with critically ill COVID-19

(A) Lollipop plot of the top 15 traits associated with critically ill COVID-19. Fold enrichment is calculated by dividing number of SNPs shared by number of SNPs expected to be shared.

(B) Plot of the pulmonary traits associated with critically ill COVID-19.

(C) Table of the five SNPs overlapping between IPF and critically ill COVID-19. Odds ratios (ORs) were calculated from the betas reported in the GWAS summary statistics. The location and functional annotation are from GENCODE on the UCSC Genome Browser and NCBI dbSNP, respectively.

(D) Miami plot of the IPF and critically ill COVID-19 GWAS highlights the five shared SNPs. Over 7.5 million SNPs were included in the IPF *meta*-GWAS summary statistics, while the top 10,000 SNPs were publicly available in the critically ill COVID-19 summary statistics from the COVID-19 Host Genetics Initiative. p values less than 1.0×10^{-40} are displayed at 40 on the y axis. Note that while an additional shared locus may be visually apparent on chromosome 3, the lead variant in the IPF GWAS is rs2292181, located at 3:44861942, while the lead variant in the COVID-19 GWAS is rs17713054, located at 3:45818159. These variants are almost 1 megabase apart and are in linkage equilibrium ($r^2 = 0.00$), and thus two independent signals. p values are $-\log_{10}$ transformed, and the red line indicates the genome-significant threshold at $p < 5.0 \times 10^{-8}$.

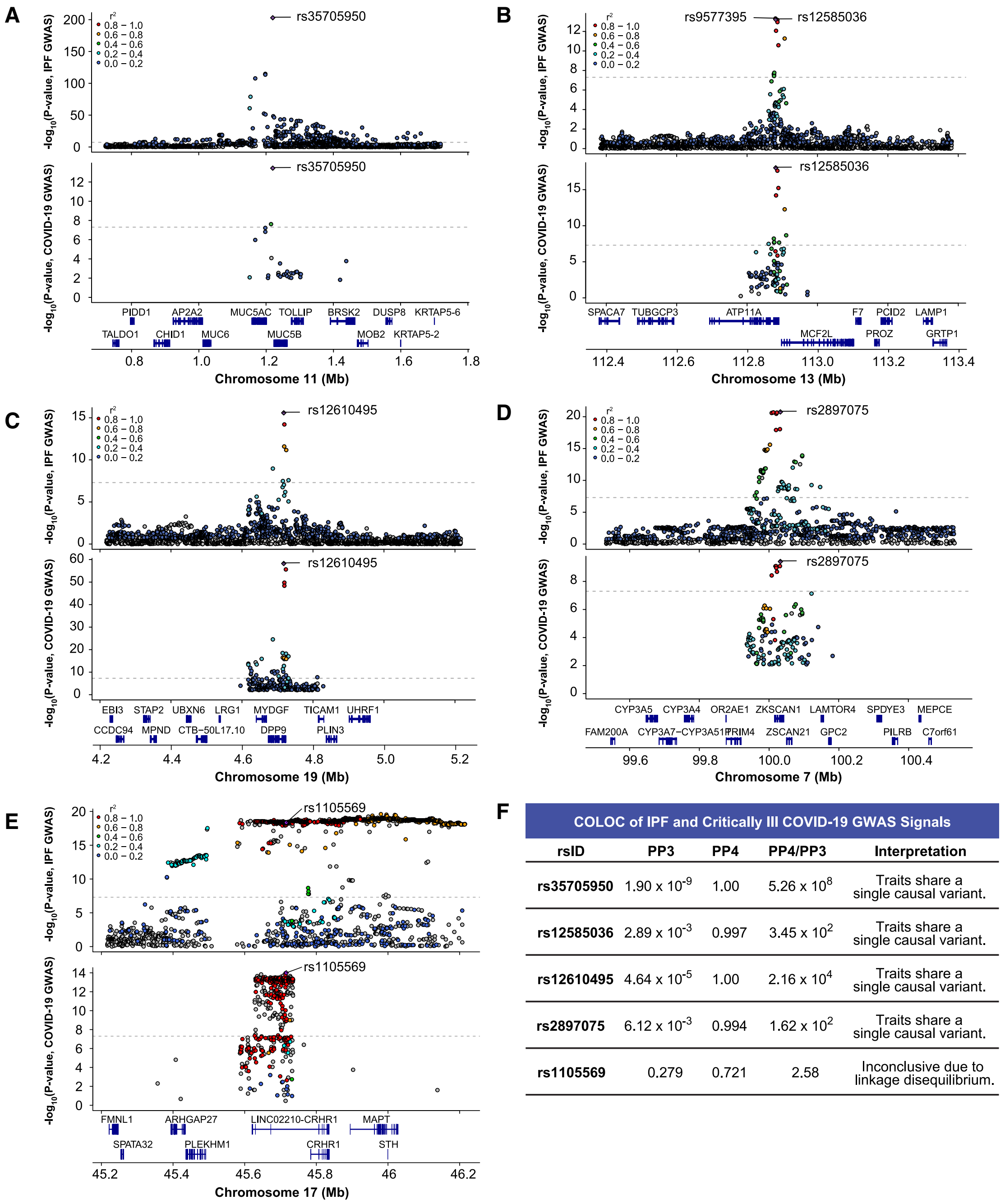

rsID	PP3	PP4	PP4/PP3	Interpretation
**rs35705950**	1.90×10^{-9}	1.00	5.26×10^{8}	Traits share a single causal variant.
**rs12585036**	2.89×10^{-3}	0.997	3.45×10^{2}	Traits share a single causal variant.
**rs12610495**	4.64×10^{-5}	1.00	2.16×10^{4}	Traits share a single causal variant.
**rs2897075**	6.12×10^{-3}	0.994	1.62×10^{2}	Traits share a single causal variant.
**rs1105569**	0.279	0.721	2.58	Inconclusive due to linkage disequilibrium.

Figure 2. IPF and critically ill COVID-19 GWAS colocalize at shared signals
(A) LocusZoom plot highlights that rs35705950 is the lead variant (purple diamond) in both disease GWAS and is located upstream of *MUC5B* on chromosome 11.
(B) rs12585036 is a top variant in the IPF GWAS and is in strong LD with the lead variant (rs9577395). rs12585036 is the lead variant in the COVID-19 GWAS and is located within an intron of *ATP11A* on chromosome 13.
(C) rs12610495 is the lead variant in both disease GWAS and is located within an intron of *DPP9* on chromosome 19.
(D) rs2897075 is the lead variant in both disease GWAS and is located within an intron of *ZKSCAN1* on chromosome 7.
(E) rs1105569 is a lead variant in both disease GWAS. rs1105569 is located within an intron of *CRHR1* and in a supergene with high linkage disequilibrium between SNPs.
(F) COLOC indicates strong colocalization between the disease GWAS signals at four of the five shared SNPs with PP4 > 0.900, and PP4/PP3 > 5.00. COLOC is inconclusive for rs1105569 due to extensive linkage disequilibrium in the region.
For (A) to (E), p values are $-\log_{10}$ transformed, and the dotted line indicates the genome-significant threshold at $p < 5.0 \times 10^{-8}$. For (A) to (E), linkage disequilibrium information for European populations was obtained from LDlink and is relative to the lead variant in each plot.

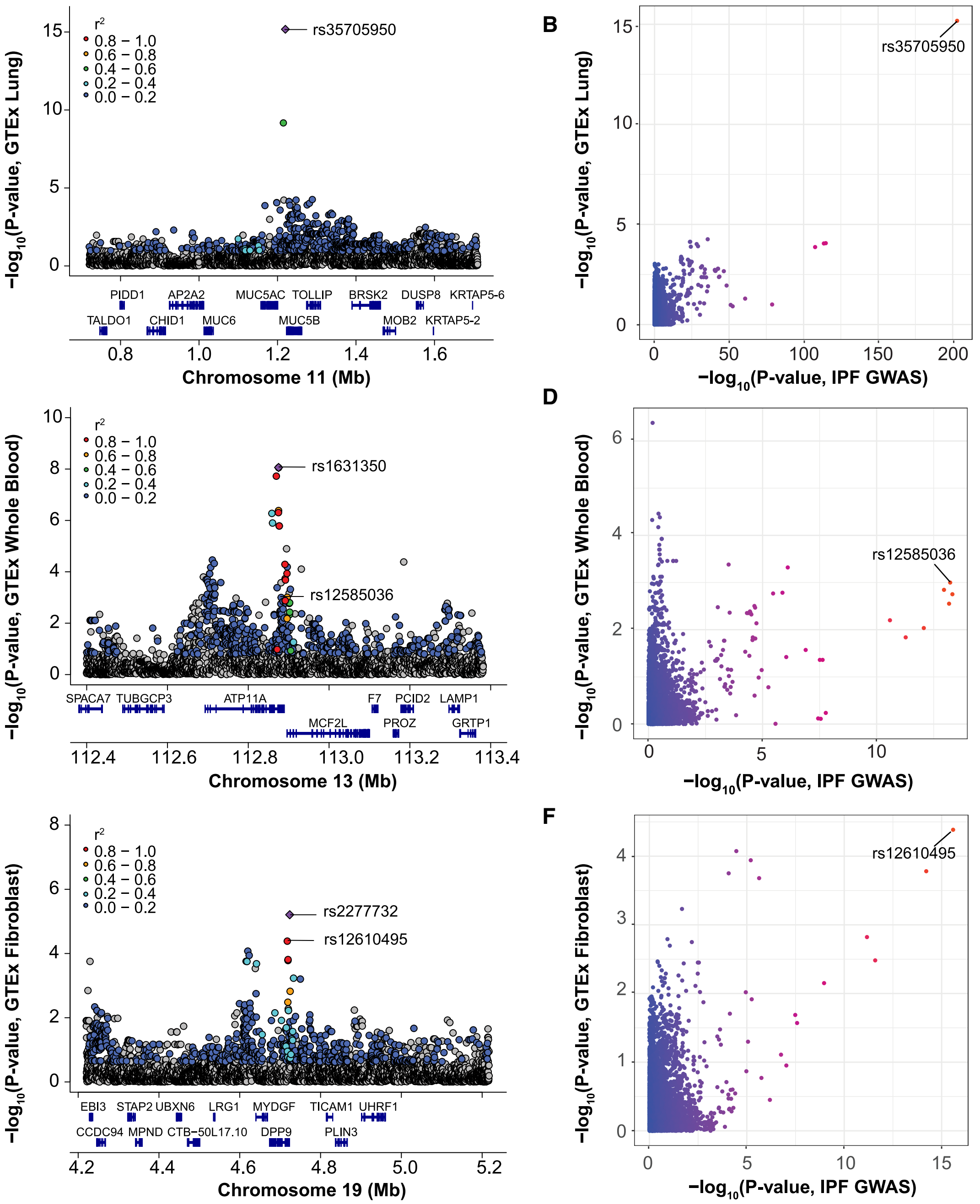

G

COLOC of GTEx eQTL and Disease GWAS Signals							
rsID	GTEx Tissue	eGene	Disease	PP3	PP4	PP4/PP3	Interpretation
rs35705950	Lung	*MUC5B*	IPF	4.12×10^{-10}	1.00	2.43×10^{9}	Traits share a single causal variant.
			COVID-19	5.26×10^{-7}	1.00	1.90×10^{6}	
rs12585036	Whole Blood	*ATP11A*	IPF	0.139	0.621	4.47	Traits likely share a single causal variant.
			COVID-19	9.70×10^{-3}	0.749	77.2	
rs12610495	Fibroblast	*DPP9*	IPF	8.27×10^{-3}	0.968	1.17×10^{2}	Traits share a single causal variant.
			COVID-19	7.85×10^{-3}	0.968	1.23×10^{2}	

Figure 3. Colocalization of disease GWAS and GTEx eQTL signals
(A) LocusZoom plot shows rs35705950 is the lead eQTL (purple diamond) for *MUC5B* in GTEx lung tissue.
(B) Comparison of $-\log_{10}$ (*p* values) from the IPF GWAS and *MUC5B*-eQTLs in GTEx lung tissue shows rs35705950 as the shared lead SNP. Note that only SNPs present in both datasets are included in the plot.
(C) rs12585036 is not the lead eQTL nor in LD with the lead eQTL (rs1631350) for *ATP11A* in GTEx whole blood.

(legend continued on next page)

signals may be due to a shared causal SNP or two independent signals driven by different causal SNPs with variable degrees of LD. To systematically reveal whether each signal was due to a single causal SNP, we performed formal colocalization testing using the critically ill COVID-19 GWAS and the IPF *meta*-GWAS (cases = 4,125, controls = 20,464). For all loci except rs1105569, the PP4 was greater than 0.900 (Figure 2F; Table S10). The exception, rs1105569 (PP4 = 0.721), is located within the 17q21.31 inversion supergene with extensive LD extending over nearly 900 kb,[32] which precluded our ability to determine whether an individual causal variant was driving the association in both diseases (Figure 2E). Overall, we determined that for the five shared loci identified by iCPAGdb, four demonstrated colocalizing signals likely driven by the same causal SNP in both critically ill COVID-19 and IPF.

Identification of causal genes for shared loci in bulk tissue

SNPs can affect a nearby gene through altering function or regulation. To speculate how each SNP contributed to gene regulation during COVID-19 and IPF, we used HaploReg,[49] a publicly available tool that annotates the putative functional impact of queried SNPs and all SNPs in LD. We first explored whether any of our SNPs of interest were in strong LD with nonsynonymous variants, altering the amino acid sequence of the protein. For rs1105569, the lead variant was in strong LD ($r^2 > 0.80$) with 18 nonsynonymous variants (missense, frameshift, and nonsense) in *CRHR1*, *SPPL2C*, and *MAPT*.[49] These three genes, and several others in 17q21.31, were previously considered important for COVID-19.[10,15,48] No nonsynonymous variants were in strong LD with the other four SNPs of interest, indicating these signals were likely due to regulatory variants.

Next, we determined if the five SNPs of interest were associated with gene expression in bulk, healthy tissue. In GTEx,[28] only rs35705950 was a conditionally independent eQTL for *MUC5B* in lung (nominal p value = 6.71×10^{-16}; beta [G/T] = 0.553). We next assessed if our SNPs of interest were in LD with conditionally independent eQTLs in any tissue (Table S3). Through GTEx and literature review, we identified 15 plausible eQTL-protein-coding eGene pairs for COLOC analysis (Table S4).

A previous study informed that rs35705950 is within an enhancer region of *MUC5B* that was differentially methylated and bound by the transcription factor FOXA2.[50] Subsequent colocalization analyses with eQTL and mQTL data from control and IPF lung tissue revealed that rs35705950-T-allele was also associated with higher methylation within a repressor region of *MUC5B* and higher *MUC5B* expression.[29] Similarly, we found that *MUC5B* was the only eGene for rs35705950 in the lung. Colocalization of the disease GWAS and *MUC5B*-eQTL signals resulted in a PP4 of 1.00, indicating that rs35705950 was the causal SNP driving both the GWAS and eQTL signals (Figures 3A and 3B; Table S9).

An LD proxy of rs12585036, rs9577395 ($r^2 = 0.99$) was a conditionally independent eQTL for *ATP11A* in tissues of unclear relevance to IPF and COVID-19 (aorta, skin, and small intestine). However, in tissues relevant to IPF and COVID-19 (EBV-transformed lymphocytes, cultured fibroblasts, lung, and whole blood), we found modest colocalization of the GWAS and *ATP11A*-eQTL signals in whole blood (PP4 = 0.621 with IPF GWAS; 0.749 with COVID-19 GWAS; Figures 3C and 3D; Table S9). However, we suspect that colocalization at this locus is due to a signal distinct from rs12585036 (nominal p value = 1.00×10^{-3}; beta [C/T] = −0.048) due to linkage equilibrium with the lead eQTL rs1631350 ($r^2 = 0.00$). In the remaining three tissues, we found weak evidence of colocalization between the GWAS and *ATP11A*-eQTL signals (PP4 < 0.408; Table S9), likely because of weakly significant eQTL p values in the locus (p values $>10^{-4}$). Additionally, the lead eQTLs were in weak LD with rs12585036 ($r^2 < 0.20$), suggesting distinct causal signals.

An LD proxy of rs12610495, rs2277732 ($r^2 = 0.95$) was a conditionally independent eQTL for *DPP9* in fibroblasts. Accordingly, the disease GWAS and *DPP9*-eQTL signals colocalized in fibroblasts (PP4 = 0.968), highlighting that rs12610495 (nominal p value = 4.12×10^{-5}; beta [A/G] = −0.065) may affect *DPP9* expression in a cell-type-specific manner (Figures 3E and 3F; Table S9). In GTEx, the rs12610495-G-allele was associated with lower *DPP9* expression in lungs and fibroblasts and was also the risk allele for both critically ill COVID-19 and IPF. In the remaining three tissues, we did not find colocalization of the GWAS and eQTL signals (PP4 < 0.225; Table S9), and the lead eQTLs were in weak LD with rs12610495 ($r^2 < 0.20$).

LD proxies of rs2897075, rs73158411, and rs13243708 were conditionally independent eQTLs in breast and

(D) Comparison of −log10 (p values) from the IPF GWAS and *ATP11A*-eQTLs in GTEx whole blood shows rs12585036 is the lead SNP for IPF but is not the lead SNP for GTEx whole blood. Multiple other SNPs in the region have a lower p value for *ATP11A* expression in GTEx whole blood. Note that the lead SNP for ATP11A expression in GTEx whole blood, rs1631350, is absent from the IPF dataset and not plotted.
(E) rs12610495 is in strong LD with the lead eQTL (rs2277732) for *DPP9* in GTEx fibroblasts.
(F) Comparison of $-\log_{10}$ (p values) from the IPF GWAS and *DPP9*-eQTLs in GTEx fibroblasts show rs12610495 as the shared lead SNP.
(G) COLOC indicates strong colocalization between the disease GWAS and *MUC5B*- and *DPP9*-eQTL signals in lung and fibroblasts, respectively, with PP4 > 0.900 and PP4/PP3 > 5.00. COLOC suggests colocalization between the COVID-19 GWAS and *ATP11A*-eQTL signals in whole blood with PP4 > 0.700 and PP4/PP3 > 5.00.
For (A) to (F), p values are −log10 transformed. For (A), (C), and (E), linkage disequilibrium information for European populations was obtained from LDlink and is relative to the lead variant in each plot.

esophageal tissue for *ZKSCAN1* and *COPS6,* respectively (Table S3). However, we found no colocalization between the disease GWAS and eQTL signals for *COPS6, TRIM4,* or *ZKSCAN1* in the tissues of interest (PP4 < 0.312; Table S9). The lead eQTLs for *COPS6, TRIM4,* or *ZKSCAN1* were in weak LD with rs2897075 ($r^2 < 0.20$).

For rs1105569, we tested nine candidate protein-coding eGenes (Table S4) but did not find clear evidence of colocalization for any eGene in the tissues of interest, which was likely due to the extensive LD in the region (Table S9). Thus, out of five shared loci, baseline bulk eQTL data in the most relevant healthy tissue (i.e., lung) only revealed a clear causal gene (*MUC5B*) for rs35705950 (Figures 3A and 3B). Our focus on the shared genetic variants between critically ill COVID-19 and IPF prompted us to broaden our search to other relevant tissues, revealing that expression of *ATP11A* in whole blood and *DPP9* in fibroblasts may be relevant for the effect of rs12585036 and rs12610495, respectively, during pathogenesis (Figure 3G). Our results highlight a mystery in the field of human genetics—lack of colocalization between GWAS and eQTL signals.[8,51,52] To address this, we next examined context-specific eQTL datasets.

rs12585036 is an eQTL for *ATP11A* in lung tissue from a cohort of primarily ex- and current smokers

The Lung eQTL Study utilized lung samples from 1,111 patients undergoing lung resection, and most (85%) were either ex-smokers or current smokers (compared with 68% of all tissue donors in GTEx; dbGaP Variable Accession: phv00408819.v1.p2).[53] We were interested in the Lung eQTL Study due to its larger sample size compared with GTEx and because smoking is a risk factor for ILDs including IPF.[54–56] The Lung eQTL Study considered age, sex, and smoking status as covariates and may reveal eQTLs that are most apparent in the context of smoking history. We hypothesized that this dataset may therefore elucidate additional eQTL signals that were not detected in GTEx (where smoking was not a covariate but might have been captured by PEER factors) and better colocalize with the IPF and COVID-19 GWAS signals. Indeed, we found that a lead eQTL for *ATP11A* in the Lung eQTL Study, rs7998551 (p value $= 7.68 \times 10^{-8}$), was in strong LD ($r^2 = 0.96$) with rs12585036 (p value $= 3.23 \times 10^{-7}$) (Figure 4A). We subsequently found strong evidence of colocalization between the disease GWAS and the *ATP11A*-eQTL signals (PP4 = 0.996 with IPF GWAS; 0.995 with COVID-19 GWAS; Figures 4B and 4C; Table S10) but weaker support for colocalization between the *ATP11A*-eQTL signals from the Lung eQTL Study and GTEx lung (PP4 = 0.344; Table S10). This demonstrated that lungs exposed to a particular environmental stressor, such as smoking, can reveal eQTLs and causal genes that would otherwise be overlooked. However, differences in sample size between the eQTL datasets (n = 515 for GTEx; n = 1,111 for the Lung eQTL Study) may also contribute to the differential detection of eQTLs; p values in GTEx for the *ATP11A* locus were greater than 5.0×10^{-3}. The rs12585036-C-allele was associated with higher *ATP11A* expression and was the risk allele in IPF but the protective allele for critically ill COVID-19. Thus, *ATP11A* appeared to have opposite effects on the pathophysiology of these two diseases.

We found no colocalization at the *MUC5B* locus since the SNP array used in the Lung eQTL study (Illumina Human1M-Duo BeadChip) did not include rs35705950, and there are no additional SNPs in LD with this SNP. We did not find colocalization at our other variants of interest.

Monocytes are a critical cell type for the eQTL effect of rs12585036

The colocalization of rs12585036 with *ATP11A* expression in lung tissue primarily from smokers revealed a plausible environmental context where an eQTL effect is revealed. However, as these data were from bulk lung tissue, it was unclear what specific cell type drove this difference. Smoking causes inflammation and infiltration of immune cells, specifically monocytes and neutrophils, into the lungs.[57–59] Therefore, we hypothesized the eQTL effect may be associated with a specific immune cell, which was also consistent with the modest colocalization we detected from bulk whole blood in GTEx (Figures 3C and 3D). BLUEPRINT,[60] a European epigenetics initiative, allowed us to interrogate *ATP11A* eQTLs in monocytes, neutrophils, and CD4+ T cells. rs12585036 was an eQTL only in monocytes (p value $= 3.58 \times 10^{-12}$ in monocytes vs. 1.38×10^{-3} in neutrophils and 3.89×10^{-2} in T cells; Figure 4D) and had the same directionality as expected from GTEx (beta (C/T) = −0.487). We found strong colocalization at this locus with the disease GWAS (PP4 = 0.994; Figures 4D–4F; Table S10). Importantly, we replicated our findings in the ImmuNexUT eQTL atlas, which utilized peripheral blood from Japanese donors to discover eQTL in 28 distinct immune cell subsets.[61] rs12585036 was an eQTL in myeloid cells (p value $= 1.10 \times 10^{-5}$ in classical monocytes vs. 1.56×10^{-2} in neutrophils and 7.56×10^{-2} in naive CD4+ T cells), and specifically, the most significant in intermediate monocytes (p value $= 8.90 \times 10^{-7}$; beta [C/T] = −0.224); Figure S1). Intermediate monocytes are characterized by surface markers ($CD14^+CD16^+$) that are canonically displayed on both classical ($CD14^+CD16^-$) and non-classical ($CD14^{dim}CD16^+$) monocytes[61] and therefore considered to be transitioning between the two states.[62] Although all three types of monocytes analyzed (classical, non-classical, and intermediate) demonstrated evidence of colocalization at rs12585036, the strongest colocalization with the disease GWAS was observed with intermediate monocytes (PP4 = 0.988 with IPF GWAS; 0.989 with COVID-19 GWAS; Figure S1; Table S10), suggesting not only cell specificity but also a relevant stage. Directionality of the signals from the Lung eQTL Study, BLUEPRINT, and ImmuNexUT were consistent with the C allele of rs12585036 being associated with higher *ATP11A* expression. Thus, our analyses used three context-specific eQTL datasets to reveal that

A

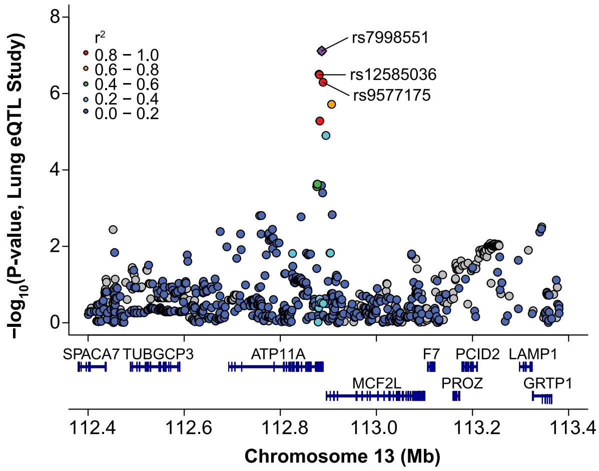

B

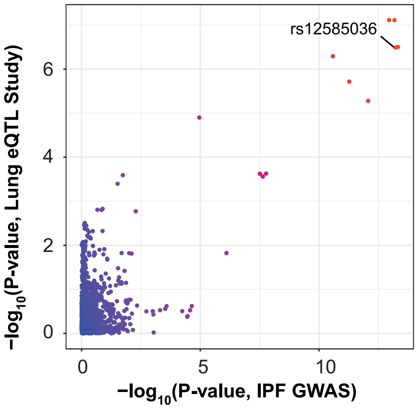

C

COLOC of the Lung eQTL Study and Disease GWAS Signals at *ATP11A*					
**rsID**	**Disease**	**PP3**	**PP4**	**PP4/PP3**	**Interpretation**
**rs12585036**	IPF	4.17×10^{-3}	0.996	2.39×10^{2}	Traits share a single causal variant.
	COVID-19	4.88×10^{-3}	0.995	2.04×10^{2}	Traits share a single causal variant.

D

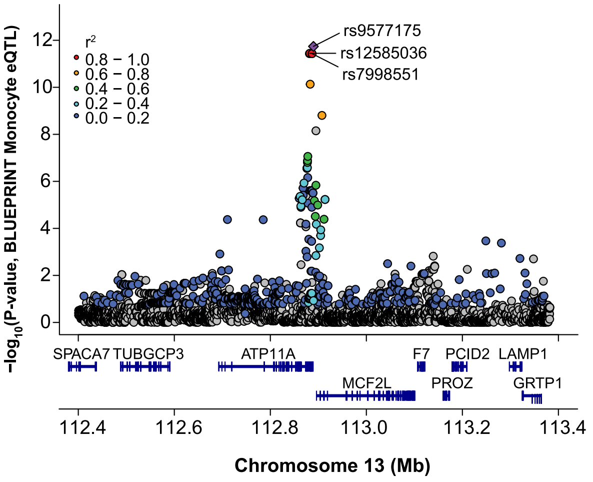

E

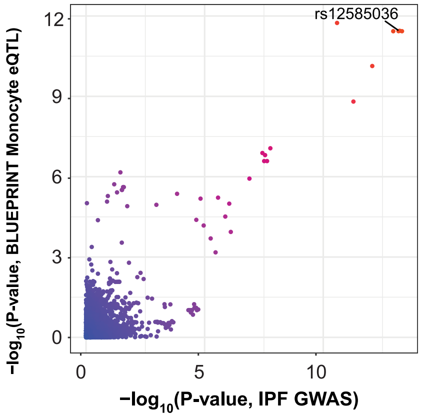

F

COLOC of the BLUEPRINT Monocyte eQTL and Disease GWAS Signals at *ATP11A*					
**rsID**	**Disease**	**PP3**	**PP4**	**PP4/PP3**	**Interpretation**
**rs12585036**	IPF	5.78×10^{-3}	0.994	1.72×10^{2}	Traits share a single causal variant.
	COVID-19	5.60×10^{-3}	0.994	1.78×10^{2}	Traits share a single causal variant.

Figure 4. Colocalization at rs12585036 reveals *ATP11A* as a causal gene in lung tissue with a history of smoking and monocytes
(A) LocusZoom plot shows that rs12585036 is a top eQTL and in strong LD with the lead eQTL (rs7998551; purple diamond) for *ATP11A* in the Lung eQTL Study, which included primarily individuals with a smoking history.
(B) Comparison of $-\log_{10}$ (p values) from the IPF GWAS and *ATP11A*-eQTLs in the Lung eQTL Study shows rs12585036 as a top shared SNP.
(C) COLOC indicates strong colocalization between the disease GWAS and the Lung eQTL Study's *ATP11A*-eQTL signals with PP4 $>$ 0.900, and PP4/PP3 $>$ 5.00.
(D) rs12585036 is a top eQTL and in strong LD with the lead eQTL (rs9577175) for *ATP11A* in monocytes in BLUEPRINT.
(E) Comparison of $-\log_{10}$(p values) from the IPF GWAS and monocytic *ATP11A*-eQTLs shows rs12585036 as a top shared SNP in BLUEPRINT.
(F) COLOC indicates strong colocalization between the disease GWAS and monocytic *ATP11A*-eQTLs signals in BLUEPRINT with PP4 $>$ 0.900 and PP4/PP3 $>$ 5.00.
For (A), (B), (D), and (E), p values are $-\log_{10}$ transformed. For (A) and (D), linkage disequilibrium information for European populations was obtained from LDlink and is relative to the lead variant in each plot.

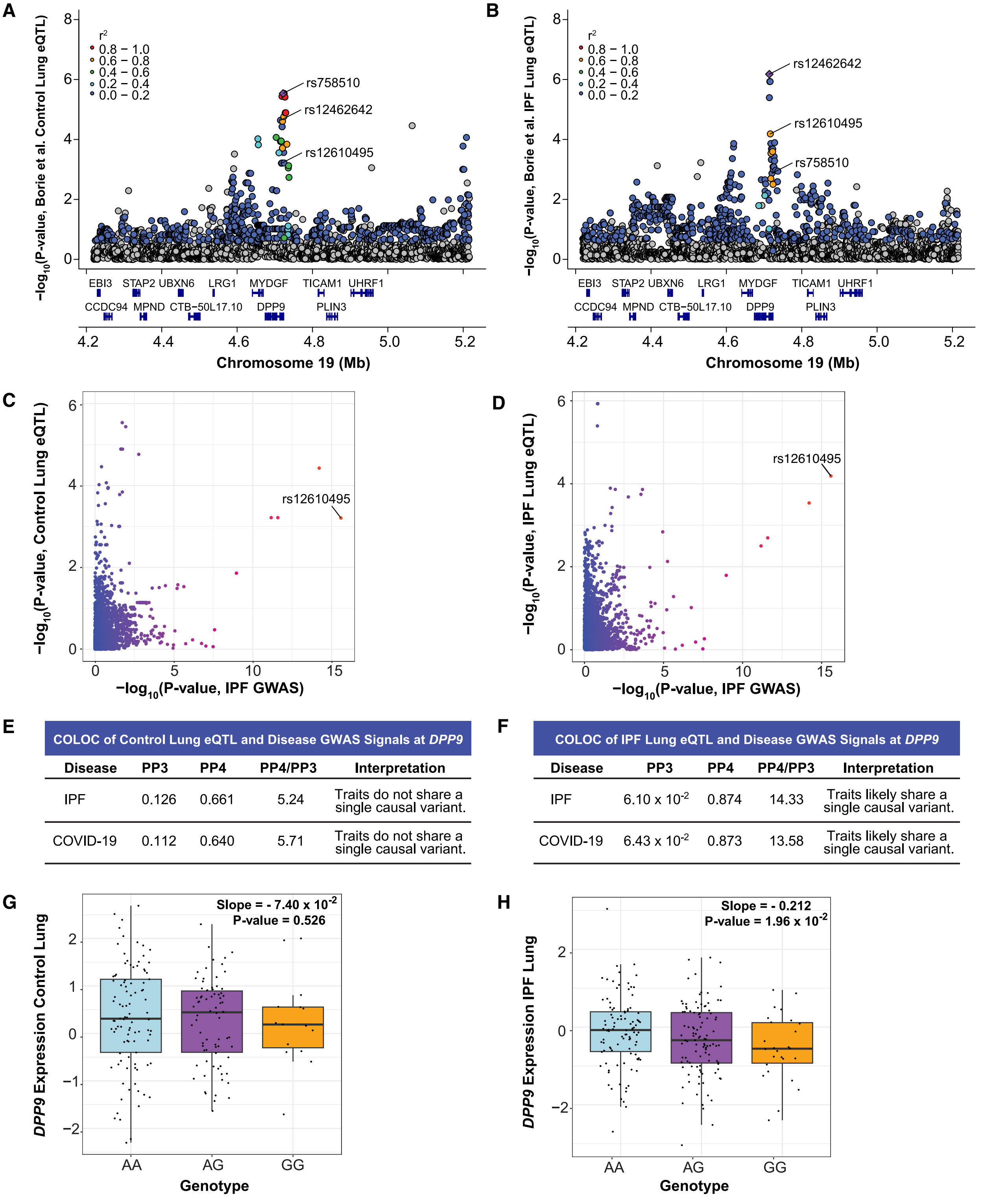

E COLOC of Control Lung eQTL and Disease GWAS Signals at *DPP9*

Disease	PP3	PP4	PP4/PP3	Interpretation
IPF	0.126	0.661	5.24	Traits do not share a single causal variant.
COVID-19	0.112	0.640	5.71	Traits do not share a single causal variant.

F COLOC of IPF Lung eQTL and Disease GWAS Signals at *DPP9*

Disease	PP3	PP4	PP4/PP3	Interpretation
IPF	6.10×10^{-2}	0.874	14.33	Traits likely share a single causal variant.
COVID-19	6.43×10^{-2}	0.873	13.58	Traits likely share a single causal variant.

Figure 5. Colocalization at rs12610495 in IPF cases reveals *DPP9* as a causal gene
(A) LocusZoom plot shows that rs12610495 is not the lead eQTL or in LD with the lead eQTL (rs758510; purple diamond) for *DPP9* in controls in the Borie et al. dataset.
(B) rs12610495 is a top eQTL and in LD with the lead eQTL (rs12462642) for *DPP9* in IPF cases.
(C) Comparison of $-\log_{10}(p$ values) from the IPF GWAS and *DPP9*-eQTLs in controls shows rs12610495 as a top SNP for IPF. Multiple other SNPs in the region have a lower p value for *DPP9* expression in controls.
(D) Comparison of $-\log_{10}(p$ values) from the IPF GWAS and *DPP9*-eQTLs in IPF cases shows rs12610495 as a top shared SNP.
(E) COLOC indicates limited evidence of colocalization between the disease GWAS and *DPP9*-eQTL signals in controls with PP4 < 0.700.

(legend continued on next page)

ATP11A was the likely causal gene associated with the IPF and COVID-19 GWAS signals at rs12585036. Further, the eQTL signal appeared to be important specifically in monocytes, possibly within the proinflammatory context of smoking.

rs12610495 is an eQTL for *DPP9* in IPF lung

We sought to determine if there was colocalization with eQTLs specifically identified in lungs from individuals with IPF. We utilized the Borie et al. eQTL dataset which included lung samples from healthy controls ($n = 188$) and individuals with IPF who also reported a more extensive smoking history ($n = 234$).[29] Borie et al. previously demonstrated colocalization of the eQTL, mQTL, and GWAS signals at rs35705950 within the *MUC5B* locus in both controls and IPF cases.[29]

We did not find evidence of colocalization between disease GWAS and control/IPF eQTL signals at rs12585036, rs2897075, and rs1105569. However, we found colocalization at rs12610495 for *DPP9* expression in IPF cases. Although the eQTL signals were in similar genomic locations for both controls and IPF cases, detailed investigation further revealed that the lead variants between the two groups were distinct signals (Figures 5A–5D). The lead variant in controls, rs758510 (p value $= 2.91 \times 10^{-6}$ from FastQTL including PEER adjustment), and the lead variant in the IPF cases, rs12462642 (6.65×10^{-7}), exhibited weak LD ($r^2 = 0.15$). In contrast, rs12462642 and rs12610495 (the lead GWAS variant; p value $= 6.52 \times 10^{-5}$; beta [A/G] $= -0.178$) were in stronger LD ($r^2 = 0.68$). COLOC demonstrated moderately strong colocalization between the disease GWAS and IPF cases *DPP9*-eQTL signals (PP4 = 0.874 with IPF GWAS; 0.873 with COVID-19 GWAS) and weaker colocalization between the GWAS and control eQTL signals (PP4 = 0.661; 0.640; Figures 5E and 5F; Table S10). Plotting *DPP9* expression by rs12610495 genotype confirmed a more robust association of this lead GWAS SNP with expression in IPF cases (slope $= -0.212$; p value $= 1.96 \times 10^{-2}$ using linear regression of pre-PEER adjusted values) compared with controls (slope $= -0.074$; p value $= 0.526$) and was consistent with the G allele being associated with lower *DPP9* expression (Figures 5G and 5H). Thus, analysis of eQTL from the GTEx fibroblasts and Borie et al. IPF lung tissue both implicate *DPP9* as the causal gene underlying rs12610495, highlighting the importance of considering cell type and disease when discovering causal eQTL. These discoveries could be related as IPF lungs are known to have high levels of activated, and potentially abnormal, fibroblasts.[63–66]

In summary, we used context-specific eQTL datasets of fibroblasts, monocytes, and lungs affected by smoking and IPF to show that *ATP11A* and *DPP9* were likely causal genes in critically ill COVID-19 and IPF. These results also revealed that *ATP11A* and *DPP9* were likely important in monocytes and fibroblasts, respectively. We also examined recent sc-eQTL datasets from Aquino et al. for COVID-19[67] and Natri et al. for IPF[68] but found no further evidence of colocalization, which could be due to low power ($n = 80$ Western European individuals in Aquino et al.; $n = 48$ controls, 66 individuals with IPF in Natri et al.).

rs12610495 is an mQTL affecting methylation of the *DPP9* promoter in IPF lungs

The stronger colocalization of GWAS and eQTL signals at rs12610495 in IPF cases suggested that there may be underlying gene regulatory differences between healthy and IPF lungs. Differences in DNA methylation can influence gene expression, and mQTL may reveal locations in the genome where patterns of DNA methylation are regulated by genetic variation.[69–71] We utilized the Borie et al. mQTL dataset, which included lung samples from healthy controls ($n = 202$) and IPF cases ($n = 345$). We found that in controls, rs12610495 (p value $= 2.14 \times 10^{-10}$; beta [A/G] $= 0.174$) was not in strong LD with rs10420225 ($r^2 = 0.25$), the lead mQTL for cg07317664 in controls. However, in cases, rs12610495 (p value $= 2.42 \times 10^{-27}$; beta [A/G] $= 0.208$) was in strong LD with rs2277732 ($r^2 = 0.95$), the lead mQTL for cg07317664 in cases (Figures 6A and 6B). We did not observe colocalization between the disease GWAS and control cg07317664-mQTL signals (PP4 $= 1.08 \times 10^{-2}$ with IPF GWAS; 7.51×10^{-3} with COVID-19 GWAS) (Figures 6C and 6E; Table S10). Like the eQTL colocalization at rs12610495, we found strong colocalization between the disease GWAS and IPF cases cg07317664-mQTL signals (PP4 = 0.979 with IPF GWAS; 0.692 with COVID-19 GWAS; Figures 6D and 6F; Table S10). Although the lack of evidence for colocalization in controls may be influenced by the smaller sample size compared with IPF cases, we suspect based on the LocusZoom and LD of rs12585036 with the lead variants (Figures 6A and 6B) that mQTL signals in the *ATP11A* locus in the controls and cases are distinct. Plotting methylation of cg07317664 by rs12610495 genotype indicated a more robust association in IPF cases (slope = 0.019; p value $= 3.20 \times 10^{-9}$ by linear regression using pre-PEER adjusted methylation values) compared with controls (slope = 0.012; p value $= 6.99 \times 10^{-4}$) (Figures 6G and 6H).

(F) COLOC indicates likely colocalization between the disease GWAS and *DPP9*-eQTL signals in IPF cases with PP4 > 0.700 and PP4/PP3 > 5.00.
(G) *DPP9* expression in controls by genotype.
(H) *DPP9* expression in IPF cases by genotype.
For (A) to (D), p values are $-\log_{10}$ transformed. For (A) and (B), linkage disequilibrium information for European populations was obtained from LDlink and is relative to the top variant in each plot. For (G) and (H), slopes and p values for genotype expression boxplots were obtained through linear regression modeling. Boxplots depict the median, interquartile range, and maximum and minimum values with outliers excluded based on 1.5x the interquartile range.

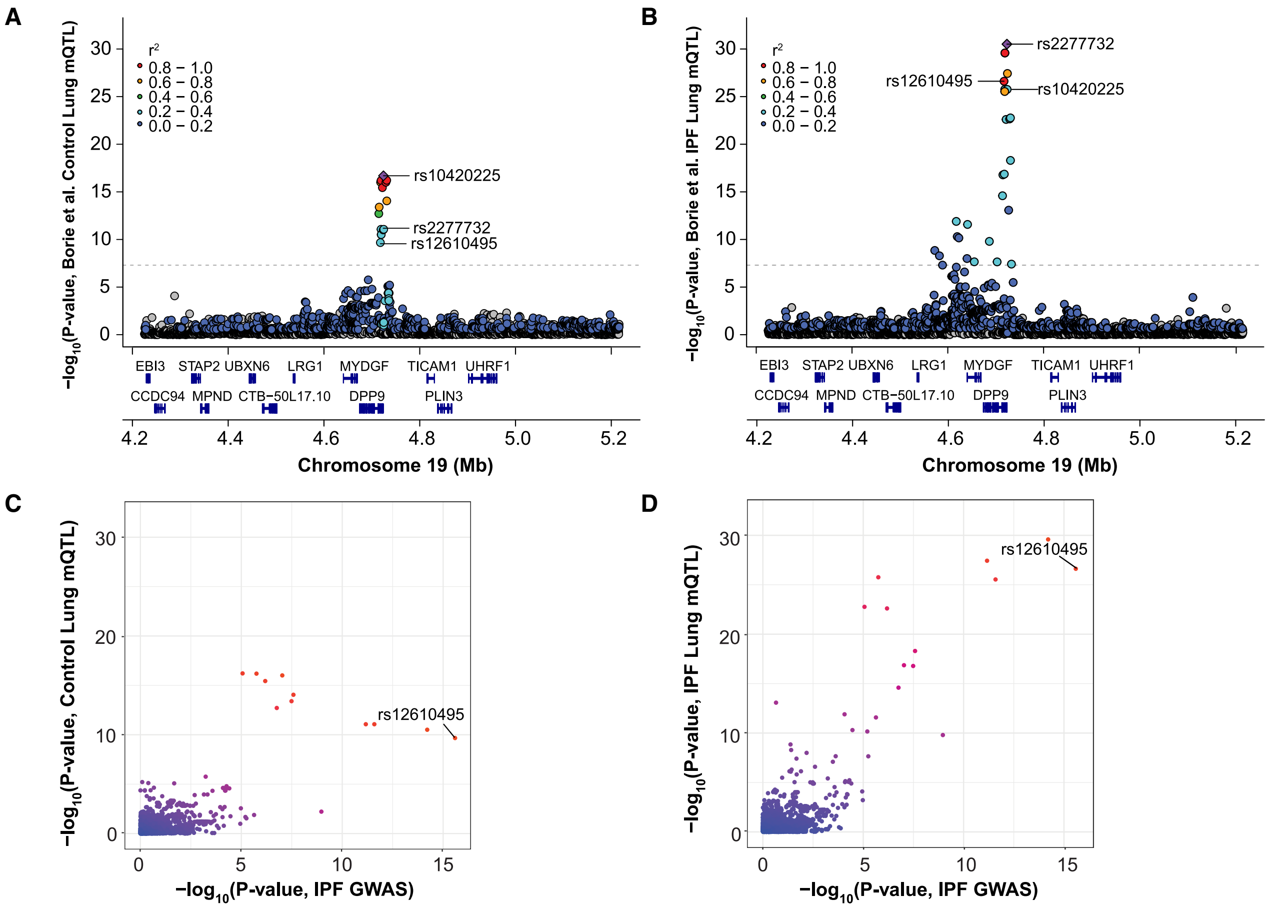

E

COLOC of Control Lung mQTL and Disease GWAS Signals at *DPP9*				
**Disease**	**PP3**	**PP4**	**PP4/PP3**	**Interpretation**
IPF	0.989	1.08 x 10^{-2}	1.09 x 10^{-2}	Traits do not share a single causal variant.
COVID-19	0.992	7.51 x 10^{-3}	7.57 x 10^{-3}	Traits do not share a single causal variant.

F

COLOC of IPF Lung mQTL and Disease GWAS Signals at *DPP9*				
**Disease**	**PP3**	**PP4**	**PP4/PP3**	**Interpretation**
IPF	2.05 x 10^{-2}	0.979	47.76	Traits share a single causal variant.
COVID-19	0.308	0.692	2.25	Traits likely share a single causal variant.

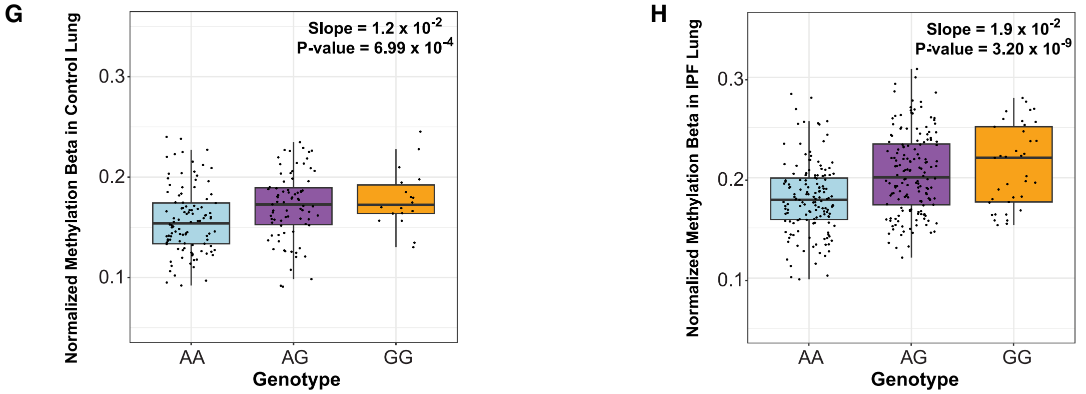

Figure 6. Colocalization at rs12610495 reveals a differentially methylated site (cg07317664) near the *DPP9* transcription start site in IPF cases

(A) LocusZoom plot shows that rs12610495 is not the lead mQTL or in LD with the lead mQTL (rs10420225; purple diamond) for cg07317664 in controls in the Borie et al. dataset.
(B) rs12610495 is a top mQTL and in LD with the lead mQTL (rs2277732) for cg07317664 in IPF cases.
(C) Comparison of $-\log_{10}(p$ values) from the IPF GWAS and cg07317664-mQTLs in controls shows rs12610495 as a top SNP for IPF. Multiple other SNPs in the region have a lower p value for methylation in controls.
(D) Comparison of $-\log_{10}(p$ values) from the IPF GWAS and cg07317664-mQTLs in IPF cases shows rs12610495 as a top shared SNP.
(E) COLOC does not support colocalization between the disease GWAS and cg07317664-mQTL signals in controls with PP4 $<$ 0.300.
(F) COLOC indicates colocalization between the disease GWAS and cg07317664-mQTL signals in cases with PP4 $>$ 0.700.

(legend continued on next page)

Cg07317664 is 7,319 base pairs upstream of the transcription start site of *DPP9,* leading us to hypothesize that methylation at this location may regulate *DPP9* expression. This hypothesis was consistent with the genetic and eQTL data as the G allele of rs12610495 was associated with greater methylation at a regulatory site of *DPP9* and decreased expression of *DPP9*, while the A allele was associated with less methylation and increased expression. Comparing the mQTL and eQTL signals in controls and cases separately demonstrated that colocalization occurred only in cases (PP4 = 0.040 in controls; 0.730 in cases; Figures 7A–7C; Table S10). Further, we found that IPF cases, regardless of rs12610495 genotype, had significantly higher methylation levels at cg07317664 (slope = 0.030; $p < 2.2 \times 10^{-16}$) and significantly lower expression of *DPP9* (slope = −0.561; p value = 9.5×10^{-9}; Figures 7D and 7E), suggesting that genotype was just one factor contributing to reduced expression in IPF and underscoring the importance of considering epigenetic regulation.

Causal genes in bulk diseased tissue

The putative causal genes we identified also demonstrated altered expression in IPF and COVID-19. Consistent with the genetic associations and as previously reported, we found that *MUC5B* expression was significantly increased in lung tissue from individuals with IPF when compared with tissue from controls in two independent studies (GEO: GSE213001 and GEO: GSE134692) (Figures S2A and S2B). In contrast, *ATP11A* and *DPP9* expression was significantly decreased in IPF lung tissue when compared with control tissue (Figures S2A and S2B). Lower *DPP9* expression in IPF lung tissue was consistent with the disease GWAS and eQTL association as the G allele of rs12610495 was associated with lower *DPP9* and increased risk of IPF. In contrast, the directionality of the observation for *ATP11A* was discordant with the GWAS and eQTL association, which suggested the C allele of rs12585036 was associated with higher *ATP11A* and increased risk of IPF. However, two reasons may reconcile this observation for *ATP11A*. First, *ATP11A* may be higher during the onset of disease and decrease in later stages, which was when most samples in these studies were collected. Later changes in *ATP11A* levels may reflect an effect of the disease on gene expression. Second, a single cell type may be underlying the genetic signal, which is confounded when looking at bulk tissue and may also be depleted during later stages of disease.

We did not observe significant differences in *MUC5B* or *DPP9* expression in lungs from patients who died of COVID-19 when compared with control lungs, which could be due to the limited number of samples in the study we examined (GEO: GSE159585; Figure S2C). We found significantly lower *ATP11A* expression in COVID-19 lungs when compared with control lungs, which was concordant with the GWAS and eQTL association as the T allele of rs12585036 was associated with low *ATP11A* and increased risk of COVID-19 (Figure S2C). However, in another study that measured gene expression in whole blood (GEO: GSE172114; Figure S2D), we found that *ATP11A* expression was higher in patients diagnosed with critically ill COVID-19 when compared with those with non-critically ill COVID-19, which could be due to an increase in myeloid lineage cells. As observed in the other datasets, *DPP9* is significantly lower in the blood of critically ill patients compared with the non-critically ill.

Altogether, these findings support the relevance of *ATP11A* and *DPP9* in IPF and COVID-19 pathogenesis. However, the directionality of how associated SNPs impact gene expression may differ in bulk diseased tissues possibly due to the impact of reverse causation of disease on gene expression, changes in cell-type proportions, and/or the lack of temporal and spatial resolution. We therefore examined scRNA-seq datasets.

Causal genes in single cell types from diseased tissue

Using a scRNA-seq dataset for IPF[39] and snRNA-seq dataset for lethal COVID-19,[40] we compared where the identified causal genes were expressed in healthy and diseased lung tissue. As differences in the expression of our identified causal genes could be due to either changes in cell type number or expression levels within a cell type, we first determined how cellular proportions differed between healthy and IPF or COVID-19 lungs. IPF lungs displayed decreases in type 1 and type 2 alveolar pneumocytes (AT1s and AT2s), and increases in other epithelial cell types (basal, ciliated, club, and goblet cells), myofibroblasts, and peribronchial vascular endothelial cells, consistent with fibrosis and reduced healthy lung tissue (Table S5). COVID-19 lungs displayed a significant increase only in macrophages but also notable increases in monocytes and fibroblasts, and decreases in AT1s, AT2s, and other airway epithelial cells (Table S6).

We subsequently determined what cell types expressed our causal genes. As expected, *MUC5B* was highly expressed in goblet cells and modestly in AT2s, other airway epithelial cells, and macrophages (Tables S7 and S8). *ATP11A* was notably expressed in AT1s, AT2s, and macrophages and modestly expressed in fibroblasts, endothelial cells, monocytes, and other airway epithelial cells (Tables S7 and S8). The expression in macrophages and

(G) Normalized methylation beta in controls by genotype.
(H) Normalized methylation beta in IPF cases by genotype.
For (A) to (D), p values are $-\log_{10}$ transformed. For (A) and (B), linkage disequilibrium information for European populations was obtained from LDlink and is relative to the top variant in each plot. For (G) and (H), slopes and p values for genotype methylation beta boxplots were obtained through linear regression modeling. Boxplots depict the median, interquartile range, and maximum and minimum values with outliers excluded based on 1.5x the interquartile range.

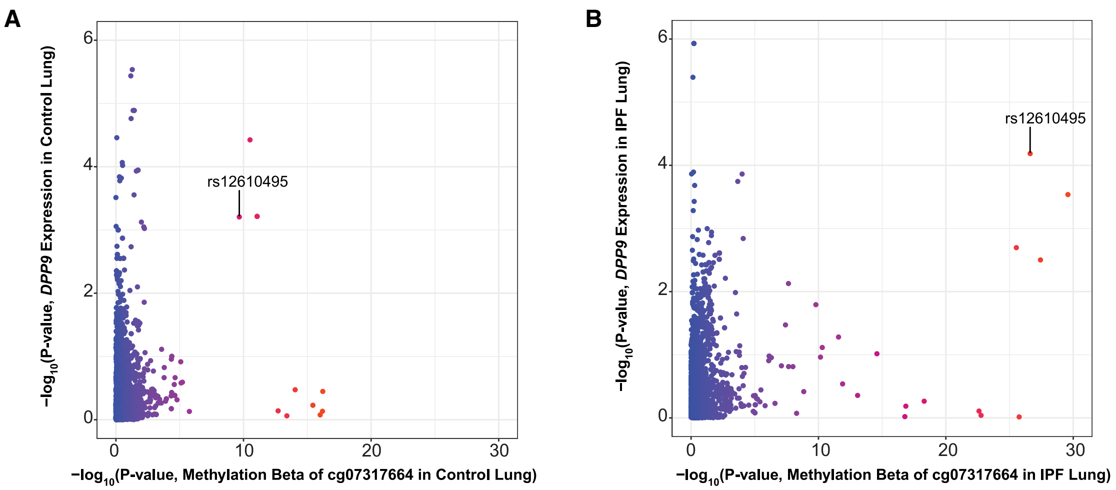

C

COLOC of cg07317664-mQTL and *DPP9*-eQTL Signals				
**Diagnosis**	**PP3**	**PP4**	**PP4/PP3**	**Interpretation**
Controls	0.354	0.040	0.114	Limited evidence of colocalization.
Cases	0.130	0.730	5.62	Traits likely share a single causal variant.

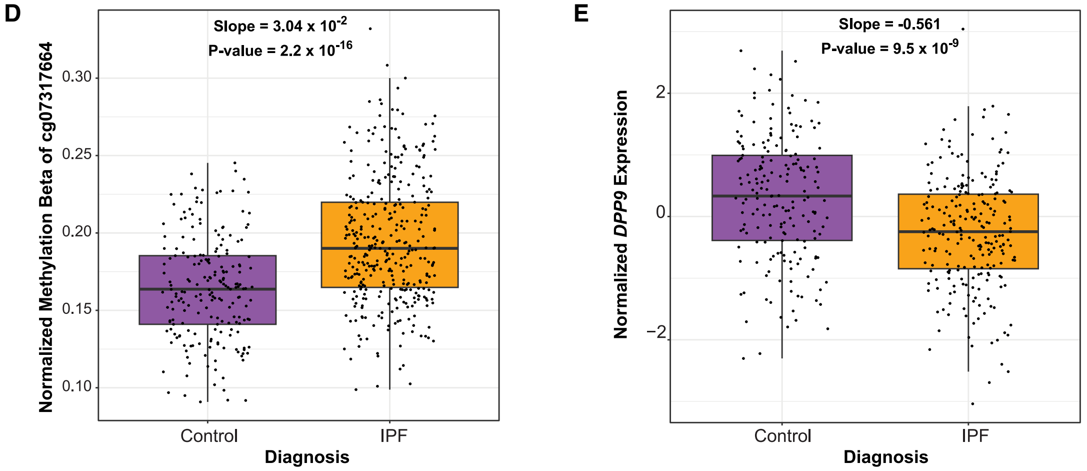

Figure 7. Colocalization at rs12610495 confirms the association between the cg07317664-mQTL and *DPP9*-eQTL signals in IPF cases
(A) Comparison of $-\log_{10}(p$ values) of cg07317664-mQTLs and *DPP9*-eQTLs in controls in the Borie et al. datasets shows that rs12610495 in not the lead SNP for either dataset.
(B) Comparison of $-\log_{10}(p$ values) in IPF cases shows rs12610495 as a top shared SNP.
(C) COLOC does not support colocalization between cg07317664-mQTL and *DPP9*-eQTL signals in controls with PP4 < 0.300 but supports colocalization in IPF cases with PP4 > 0.700.
(D) Normalized methylation beta in lung by diagnosis.
(E) *DPP9* expression in lung by diagnosis.
For (D) and (E), p values for boxplots were calculated using an unpaired t-test. Boxplots depict the median, interquartile range, and maximum and minimum values with outliers excluded based on 1.5x the interquartile range.

monocytes was particularly intriguing given the colocalization of monocyte eQTLs and GWAS signals at the *ATP11A* locus. *DPP9* was notably expressed in AT1s, AT2s, macrophages, and fibroblasts (Tables S7 and S8).

Next, we examined differences in causal gene expression during IPF and COVID-19 in single cell types. Concordant with the GWAS and eQTL directionality, we found that *MUC5B* was significantly increased during IPF in goblet cells, AT2s, other airway epithelial cells, and macrophages (Figure 8A; Table S7). In COVID-19, we found decreased, although statistically nonsignificant, *MUC5B* in airway epithelial cells, which includes goblet cells (Figure 8B; Table S8), consistent with our genetic associations having opposite effects in these two diseases.

We hypothesized that *ATP11A* would be increased in epithelial cells and/or myeloid lineage cells based on the GWAS and eQTL colocalization in lung tissue primarily from smokers and in monocytes. However, we found

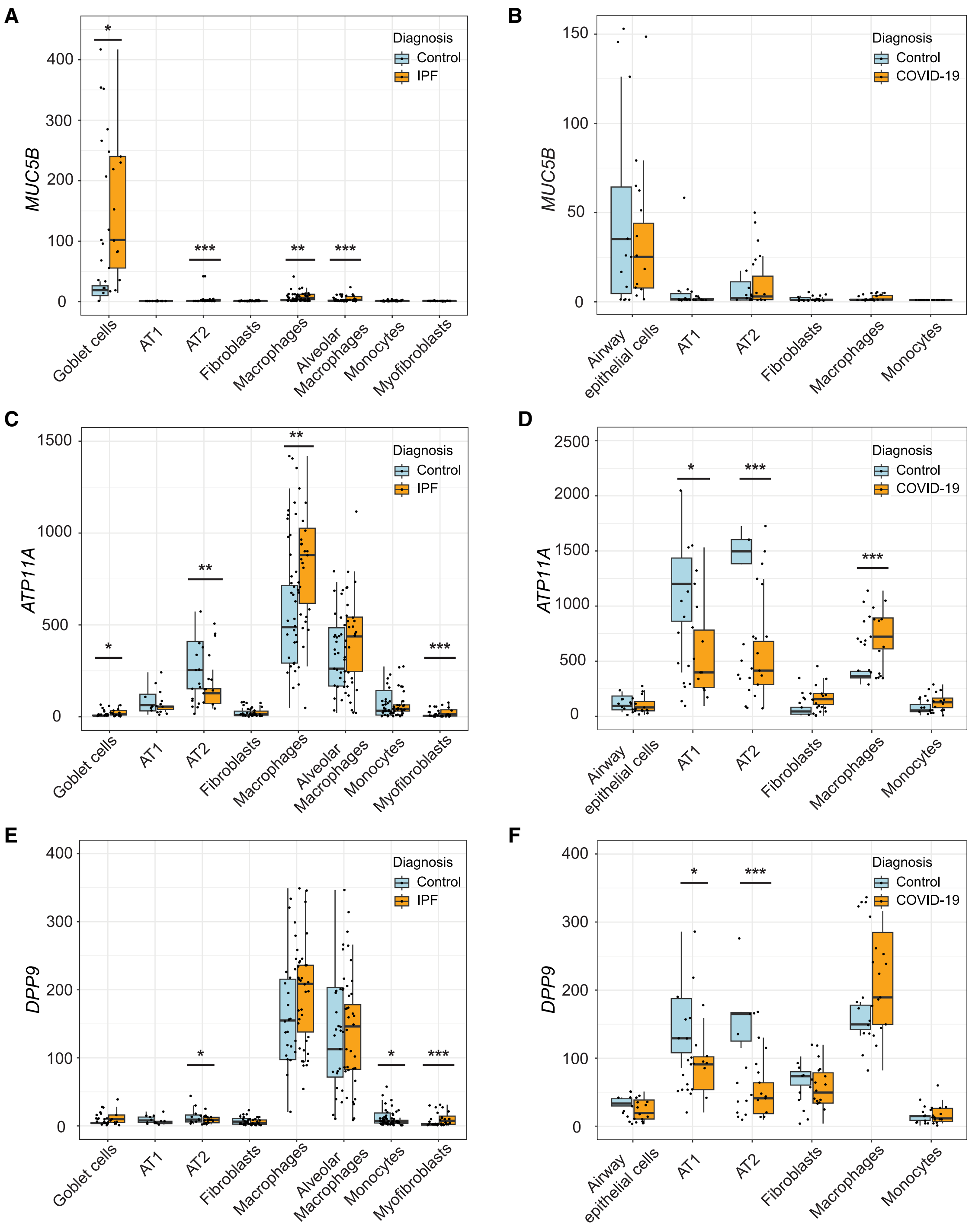

Figure 8. Assessment of causal genes at the single-cell resolution in control and diseased tissue

(A, C, and E) Boxplots depict normalized expression of (A) *MUC5B*, (C) *ATP11A*, and (E) *DPP9* in an IPF single-cell RNA sequencing study by Adams et al. that included lung tissue from controls ($n = 28$) and IPF patients ($n = 32$).

(B, D, and F) Boxplots depict normalized expression of (B) *MUC5B*, (D) *ATP11A*, and (F) *DPP9* in a lethal COVID-19 single-nucleus RNA sequencing study by Melms et al. that included lung tissue from controls ($n = 7$) and patients who died of COVID-19 ($n = 19$). p values were calculated using a Wald test and adjusted for multiple comparisons using the Benjamini-Hochberg method (*$p$adj < 0.05; **$p$adj < 0.001; ***$p$adj < 0.0001).

Boxplots depict the median, interquartile range, and maximum and minimum values with outliers excluded based on 1.5x the interquartile range.

significantly lower expression in AT2s (Figure 8C; Table S7), which may be due to the depletion of epithelial cells during IPF. Consistent with our hypothesis, *ATP11A* was significantly increased during IPF in macrophages, a large proportion of which are derived from monocytes recruited to the lung during IPF,[72] as well as in other airway epithelial cells

and myofibroblasts, which are a key driver of fibrosis (Figure 8C; Table S7).[63] During COVID-19, *ATP11A* was significantly decreased in AT1s, AT2s, other airway epithelial cells, concordant with the GWAS and eQTL data (Figure 8D; Table S8).

We hypothesized that *DPP9* would be decreased during IPF and COVID-19 in fibroblasts and other cell types that increase in proportion during IPF based on our GWAS and eQTL colocalization in fibroblasts and IPF lung. *DPP9* was significantly decreased in AT2s and monocytes during IPF (Figure 8E; Table S7). However, we found that *DPP9* was significantly increased in myofibroblasts during IPF, which may be due to the hyperproliferation of this cell type during IPF (Figure 8E; Table S7). During COVID-19, we found that *DPP9* was significantly decreased in AT1s and AT2s (Figure 8F; Table S8).

Discussion

GWASs provide a powerful approach to gather human genetic evidence to increase success in drug development, but such efforts require the identification of the underlying causal genes.[1] Molecular QTL studies were designed to bridge the gap between GWAS variants and causal genes, revealing molecular effects on gene expression, splicing, and methylation. However, studies have found limited colocalization between eQTL and human disease GWAS signals. This has been speculated to be due to multiple factors including lack of power in detecting GWAS and eQTL signals; inappropriate context with regard to cell type, developmental stage, and response; nonlinear relationships between eQTL effect and phenotype; overlap of eQTL signals due to multiple signals; and strong LD between variants limiting deconvolution of causality (e.g., rs1105569 within the 17q21.31 inversion supergene). Here, we found evidence that consideration of cell type and disease can improve colocalization of GWAS and QTL signals to reveal causal genes and where they may function during pathogenesis. Whereas eQTLs from healthy lung revealed *MUC5B* as the causal gene underlying the association of rs35705950 with critically ill COVID-19 and IPF, *MUC5B* may be atypical in that its expression is largely restricted to mucus-secreting cells and the effect size of the eQTL is large,[29] making accurate mapping of this eQTL feasible with smaller numbers and in bulk tissue. Examination of eQTL data from isolated cell types and lung disease states was necessary to reveal *DPP9* and *ATP11A* as two additional causal genes. Thus, well-powered tissue- and disease-specific eQTL datasets will continue to reveal additional causal genes for human diseases.

For each of the five shared loci between critically ill COVID-19 and IPF, identification of the likely causal gene leads to testable hypotheses for how each gene impacts these two diseases. This has been demonstrated for the *MUC5B* locus in IPF. The variant rs35705950 is within an enhancer that is differentially regulated and binds the transcription factor FOXA2 to regulate *MUC5B* expression.[50] Additional colocalization studies at rs35705950 between IPF GWAS, control and IPF eQTL, and mQTL signals suggested that higher methylation at a repressor region further contributes to increased *MUC5B* expression in cases of IPF.[29] *In vivo* studies have functionally demonstrated that excessive MUC5B production impairs mucociliary clearance and increases the fibroproliferative response, while reducing mucin production may ameliorate fibrosis.[73] Though increased mucus plugging was observed in COVID-19 lung autopsies,[74] the same allele associated with increased *MUC5B* is protective against severe COVID-19, suggesting increased mucus production in this acute context is protective. The opposite risk alleles of *MUC5B* (and *ATP11A*) for IPF and critically ill COVID-19 also underscore that though these two diseases share five of the same genetic determinants, the underlying pathophysiology is unique—PCPF after critically ill COVID-19 is not simply IPF due to a known etiology. For the remaining four overlapping loci, our identification of causal genes now informs hypotheses that may elucidate how these variants potentially affect COVID-19 and IPF.

The causal gene associated with rs2897075 has been difficult to identify due to lack of colocalization between GWAS and eQTL signals. We speculate that the most likely causal genes are *TRIM4* and *ZKSCAN1*. rs2897075 is an eQTL for *TRIM4* in several tissues, including the lung, and decreased expression is associated with the risk allele of critically ill COVID-19 and IPF. TRIM4 has been reported to polyubiquitinate viral pattern recognition receptors including RIG-I and MDA5 and promote type I interferon production during Sendai virus[75] and SARS-CoV-2[76] infections, respectively. rs2897075 is also an eQTL for *ZKSCAN1* in fibroblasts, and decreased expression is associated with the risk allele of IPF and critically ill COVID-19. Interestingly, *ZKSCAN1* mRNA can be backspliced into a circular RNA (circRNA), a type of noncoding RNA that can act as sponges for microRNA and proteins and may contribute to fibrosis.[77,78] Whether rs2897075 is associated with levels of *ZKSCAN1* circRNA is unknown, though both *ZKSCAN1* and *circZKSCAN1* have been implicated in liver fibrosis.[79] Although several studies have reported rs2897075 as a genome-wide significant hit in IPF GWASs, its mechanism and phenotypic effect has not been reported to our knowledge. Importantly, we noted that although rs2897075 was not an eQTL in the Borie et al. eQTL dataset, a variant in strong LD rs6963345 ($r^2 = 1.00$) was associated with numerous mQTLs, suggesting that this SNP may influence DNA methylation.[29]

The directionality of the rs12585036 alleles indicates that increased *ATP11A* expression is associated with increased risk of IPF while decreased expression is associated with increased risk of critically ill COVID-19. Our colocalization data suggest that the association at the *ATP11A* locus may be due to expression in monocytes and derived cells in the lung. Cycles of alveolar damage, recruitment of monocytes, and their differentiation into

monocyte-derived alveolar macrophages occur repeatedly during IPF.[72] Specifically, M2 macrophages promote fibrosis, possibly through secretion of TGF-beta[80] and other profibrotic factors.[72] ATP11A, or ATPase Phospholipid Transporting 11A, is a member of the P4-ATPase family of lipid flippases, responsible for translocating aminophospholipids on the cellular membrane.[81,82] We propose two mechanisms that could explain ATP11A's involvement in the pathogenesis of IPF and COVID-19 (Figure S3A). First, ATP11A is cleaved during apoptosis and results in phosphatidylserine's translocation to the outer leaflet of the cell membrane, which is an "eat me" signal during efferocytosis.[81–84] Increased apoptosis of epithelial cells and resistance to apoptosis in myofibroblasts are hallmarks of IPF, correlating with aberrant collagen deposition.[85,86] During IPF, we speculate that increased ATP11A may contribute to impaired efferocytosis, an observation reported in murine models[87] and IPF patient samples.[88] Second, ATP11A has also been reported to promote internalization of toll-like receptor 4 (TLR4), which is highly expressed on both alveolar pneumocytes and myeloid lineage cells, preventing hypersecretion of proinflammatory cytokines after stimulation with lipopolysaccharide.[89] This may skew macrophages toward anti-inflammatory, profibrotic M2 polarization and is supported by our findings that ATP11A is most significant as an eQTL in intermediate monocytes ($CD14^{+}CD16^{+}$), which are considered to be between the classical ($CD14^{+}$ $CD16^{-}$) and non-classical ($CD14^{dim}CD16^{+}$) monocytes.[62] Additionally, TLR4 signaling contributes to proper AT2 renewal and lung repair.[90] In COVID-19, the ectodomain of the SARS-CoV-2 spike protein has been reported to stimulate TLR4,[91,92] though other reports suggest lipopolysaccharide or other contaminants may be driving immune activation.[93–96] Regardless of whether SARS-CoV-2 directly stimulates TLR4, without adequate internalization of TLR4 by ATP11A, downstream immune signaling may be overactivated, leading to hypersecretion of proinflammatory cytokines. Thus, we propose that ATP11A may affect IPF and COVID-19 by functioning in both epithelial and myeloid cells and by impairing cell death and immune pathways. Additionally, the opposite effects of rs12585036 and rs35705950 during IPF and COVID-19 highlight the trade-offs of specific genetic variants in the face of different threats.

Colocalization of disease GWAS and eQTL signals indicates that decreased *DPP9* expression is associated with increased risk of COVID-19 and IPF and that fibroblasts may drive the association at least in IPF lung. DPP9, DipeptidylPeptidase 9, is a serine peptidase that is in the same family as DPP4, which is well-known as a target of hypoglycemic drugs and as a receptor for the coronavirus causing Middle Eastern respiratory syndrome. DPP9 helps maintain the inactive conformation of the NLRP1 inflammasome, inhibiting activation and downstream pyroptosis.[97–99] In addition to regulating inflammasome activity, DPP9 also regulates survival, proliferation, migration, and adhesion, including in dermal fibroblasts.[100] Interestingly, fibroblast activating protein (FAP) belongs to the same serine protease subfamily as DPP9. However, unlike the other DPP proteins, FAP is largely expressed by cancerous cells, inducing epithelial-mesenchymal transition. In IPF, FAP is specifically localized to fibrotic foci and not in normal tissue.[101] DPP9 interacts with FAP directly in oral squamous cell carcinoma, and *DPP9* overexpression can inhibit the epithelial-mesenchymal transition activity induced by FAP.[102] We previously reported that based on transcriptomics from patients infected with COVID-19 that *DPP9* decreased concordantly with resolution of infection, and therefore, may be dampening the inflammatory response.[12] We suspect that lower DPP9 may exacerbate pathogenesis of IPF and COVID-19 by preventing excess NLRP1-mediated inflammation and weakening cell-to-cell adhesion, promoting a dysregulated cycle of inflammation and fibrosis (Figure S3B).

While the evidence we have presented suggests that variants in *ATP11A* and *DPP9* impact COVID-19 and IPF pathogenesis by altering expression of these genes, recent evidence from Nakanishi et al. demonstrated that the sQTL data for these loci also colocalize with severe COVID-19.[103] Future studies will be necessary to understand the effect of these risk variants on splicing, but we note that each sQTL is predicted to only impact a single non-canonical isoform based on Ensembl annotation. The isoform associated with *DPP9* (ENST00000599248) is not protein-coding; the associated isoform for *ATP11A* (ENST00000415301) is highly truncated. Thus, *ATP11A* and *DPP9* demonstrate a new "problem" in human genetics; the same human disease signal may colocalize with multiple molecular QTL signals, expanding the list of possible causal genes that underlie a GWAS signal. eQTL and sQTL data may also demonstrate two consequences of the same SNP as altering splicing can certainly impact overall gene expression.

There are some limitations to our study, which can be further addressed in future work. We used p values for COLOC analysis as beta coefficients and variance of beta were not available in the summary statistics of several eQTL studies. Although p values are routinely used in COLOC, analysis with beta coefficients, representing effect sizes, may be less sensitive to differences in sample size between the paired traits. While GWAS summary statistics now include thousands of participants, context-specific eQTL studies are novel datasets often with limited sample sizes. Additionally, the COLOC framework assumes a single causal variant per trait. We investigated the presence of multiple causal variants by performing COLOC and visualizing LD through LocusZoom plots. However, SuSiE, a newer iteration of COLOC, considers the hypothesis that one locus may have multiple causal variants by accounting for LD structure between the SNPs.[7] Further, although we deeply focused on eQTLs and also considered mQTLs, future studies incorporating more information on context-specific sQTLs, pQTLs, and other molecular QTLs

will be critical for revealing biologically meaningful causal variants and their potential mechanisms of action. Finally, while statistical colocalization provides strong evidence for causality, the importance of these identified causal genes can now be rigorously tested through experimental methods in the appropriate cell types and context.

Identifying shared genetic associations and their causal genes can reveal new insight into the pathogeneses of COVID-19 and IPF that could spur further therapeutic development. Indeed, MUC5B has been proposed as a target for IPF treatment since the genetic association was first reported.[104] For the causal genes with new evidence presented here, *DPP9* may be particularly attractive, and inhibitors in development have been speculated to be therapeutic for pulmonary fibrosis.[105] However, for treatment of COVID-19 and IPF, increasing *DPP9* expression or activity seems to be the desired outcome. It remains to be tested if recombinant DPP9 or increasing expression in fibroblasts is beneficial in critically ill COVID-19 or IPF.

Data and code availability

R code for the analyses is available on GitHub at: https://github.com/tdalapati/covidipfeqtl.

Acknowledgments

T.D., L.W., A.G.J., and D.C.K. were supported by R01AI118903 and R01AI170089. T.D. was supported by a TriCEM Graduate Student Award and the Gertrude B. Elion Mentored Medical Student Research Award. We thank Dr. Richard J. Allen and colleagues for sharing the IPF meta-GWAS summary statistics.

Declaration of interests

The authors declare no competing interests.

Web resources

COVID19-hg GWAS meta-analyses round 7: https://www.covid19hg.org/results/r7/.

iCPAGdb: http://cpag.oit.duke.edu.

GTEx: https://www.gtexportal.org.

GTEx v8, Tissue-Specific All SNP Gene Association: https://www.gtexportal.org/home/downloads/adult-gtex/qtl.

GTEx v8 Smoking Status (dbGaP Variable Accession: phv00408819.v1.p2): https://www.ncbi.nlm.nih.gov/projects/gap/cgi-bin/variable.cgi?study_id=phs000424.v8.p2&phv=408819.

HaploReg v4.2: https://pubs.broadinstitute.org/mammals/haploreg/haploreg.php.

ImmuNexUT: https://www.immunexut.org.

LDlink: https://ldlink.nih.gov.

R package "locuszoomr": https://cran.r-project.org/web/packages/locuszoomr/.

Single Cell Portal: https://singlecell.broadinstitute.org.

Supplemental information

Supplemental information can be found online at https://doi.org/10.1016/j.xhgg.2025.100410.

Received: July 15, 2024
Accepted: January 22, 2025

References

1. Nelson, M.R., Tipney, H., Painter, J.L., Shen, J., Nicoletti, P., Shen, Y., Floratos, A., Sham, P.C., Li, M.J., Wang, J., et al. (2015). The support of human genetic evidence for approved drug indications. Nat. Genet. *47*, 856–860. https://doi.org/10.1038/ng.3314.
2. Ochoa, D., Karim, M., Ghoussaini, M., Hulcoop, D.G., McDonagh, E.M., and Dunham, I. (2022). Human genetics evidence supports two-thirds of the 2021 FDA-approved drugs. Nat. Rev. Drug Discov. *21*, 551. https://doi.org/10.1038/d41573-022-00120-3.
3. Gratten, J., and Visscher, P.M. (2016). Genetic pleiotropy in complex traits and diseases: implications for genomic medicine. Genome Med. *8*, 78. https://doi.org/10.1186/s13073-016-0332-x.
4. Watanabe, K., Stringer, S., Frei, O., Umićević Mirkov, M., de Leeuw, C., Polderman, T.J.C., van der Sluis, S., Andreassen, O.A., Neale, B.M., and Posthuma, D. (2019). A global overview of pleiotropy and genetic architecture in complex traits. Nat. Genet. *51*, 1339–1348. https://doi.org/10.1038/s41588-019-0481-0.
5. Hormozdiari, F., van de Bunt, M., Segrè, A.V., Li, X., Joo, J.W.J., Bilow, M., Sul, J.H., Sankararaman, S., Pasaniuc, B., and Eskin, E. (2016). Colocalization of GWAS and eQTL Signals Detects Target Genes. Am. J. Hum. Genet. *99*, 1245–1260. https://doi.org/10.1016/j.ajhg.2016.10.003.
6. Giambartolomei, C., Vukcevic, D., Schadt, E.E., Franke, L., Hingorani, A.D., Wallace, C., and Plagnol, V. (2014). Bayesian test for colocalisation between pairs of genetic association studies using summary statistics. PLoS Genet. *10*, e1004383. https://doi.org/10.1371/journal.pgen.1004383.
7. Wallace, C. (2021). A more accurate method for colocalisation analysis allowing for multiple causal variants. PLoS Genet. *17*, e1009440. https://doi.org/10.1371/journal.pgen.1009440.
8. Mostafavi, H., Spence, J.P., Naqvi, S., and Pritchard, J.K. (2023). Systematic differences in discovery of genetic effects on gene expression and complex traits. Nat. Genet. *55*, 1866–1875. https://doi.org/10.1038/s41588-023-01529-1.
9. Pan, S., Kang, H., Liu, X., Li, S., Yang, P., Wu, M., Yuan, N., Lin, S., Zheng, Q., and Jia, P. (2024). COLOCdb: a comprehensive resource for multi-model colocalization of complex traits. Nucleic Acids Res. *52*. D871-d881. https://doi.org/10.1093/nar/gkad939.
10. Kanai, M., Andrews, S.J., Cordioli, M., Stevens, C., Neale, B.M., Daly, M., Ganna, A., Pathak, G.A., Iwasaki, A., Karjalainen, J., et al. (2023). A second update on mapping the human genetic architecture of COVID-19. Nature *621*. E7-e26. https://doi.org/10.1038/s41586-023-06355-3.
11. Wang, L., Oehlers, S.H., Espenschied, S.T., Rawls, J.F., Tobin, D.M., and Ko, D.C. (2015). CPAG: software for leveraging pleiotropy in GWAS to reveal similarity between human traits links plasma fatty acids and intestinal inflammation. Genome Biol. *16*, 190. https://doi.org/10.1186/s13059-015-0722-1.
12. Wang, L., Balmat, T.J., Antonia, A.L., Constantine, F.J., Henao, R., Burke, T.W., Ingham, A., McClain, M.T., Tsalik, E.L., Ko, E.R., et al. (2021). An atlas connecting shared

genetic architecture of human diseases and molecular phenotypes provides insight into COVID-19 susceptibility. Genome Med. *13*, 83. https://doi.org/10.1186/s13073-021-00904-z.
13. Severe Covid-19 GWAS Group (2020). Genomewide Association Study of Severe Covid-19 with Respiratory Failure. N. Engl. J. Med. *383*, 1522–1534. https://doi.org/10.1056/NEJMoa2020283.
14. Allen, R.J., Guillen-Guio, B., Oldham, J.M., Ma, S.F., Dressen, A., Paynton, M.L., Kraven, L.M., Obeidat, M., Li, X., Ng, M., et al. (2020). Genome-Wide Association Study of Susceptibility to Idiopathic Pulmonary Fibrosis. Am. J. Respir. Crit. Care Med. *201*, 564–574. https://doi.org/10.1164/rccm.201905-1017OC.
15. Degenhardt, F., Ellinghaus, D., Juzenas, S., Lerga-Jaso, J., Wendorff, M., Maya-Miles, D., Uellendahl-Werth, F., ElAbd, H., Rühlemann, M.C., Arora, J., et al. (2022). Detailed stratified GWAS analysis for severe COVID-19 in four European populations. Hum. Mol. Genet. *31*, 3945–3966. https://doi.org/10.1093/hmg/ddac158.
16. Kousathanas, A., Pairo-Castineira, E., Rawlik, K., Stuckey, A., Odhams, C.A., Walker, S., Russell, C.D., Malinauskas, T., Wu, Y., Millar, J., et al. (2022). Whole-genome sequencing reveals host factors underlying critical COVID-19. Nature *607*, 97–103. https://doi.org/10.1038/s41586-022-04576-6.
17. Pairo-Castineira, E., Clohisey, S., Klaric, L., Bretherick, A.D., Rawlik, K., Pasko, D., Walker, S., Parkinson, N., Fourman, M.H., Russell, C.D., et al. (2021). Genetic mechanisms of critical illness in COVID-19. Nature *591*, 92–98. https://doi.org/10.1038/s41586-020-03065-y.
18. Allen, R.J., Guillen-Guio, B., Croot, E., Kraven, L.M., Moss, S., Stewart, I., Jenkins, R.G., and Wain, L.V. (2022). Genetic overlap between idiopathic pulmonary fibrosis and COVID-19. Eur. Respir. J. *60*, 2103132. https://doi.org/10.1183/13993003.03132-2021.
19. Fadista, J., Kraven, L.M., Karjalainen, J., Andrews, S.J., Geller, F., COVID-19 Host Genetics Initiative, Baillie, J.K., Wain, L.V., Jenkins, R.G., and Feenstra, B. (2021). Shared genetic etiology between idiopathic pulmonary fibrosis and COVID-19 severity. EBioMedicine *65*, 103277. https://doi.org/10.1016/j.ebiom.2021.103277.
20. Verma, A., Minnier, J., Wan, E.S., Huffman, J.E., Gao, L., Joseph, J., Ho, Y.L., Wu, W.C., Cho, K., Gorman, B.R., et al. (2022). A MUC5B gene polymorphism, rs35705950-T, confers protective effects against COVID-19 hospitalization but not severe disease or mortality. Am. J. Respir. Crit. Care Med. *206*, 1220–1229. https://doi.org/10.1164/rccm.202109-2166OC.
21. Davis, H.E., McCorkell, L., Vogel, J.M., and Topol, E.J. (2023). Long COVID: major findings, mechanisms and recommendations. Nat. Rev. Microbiol. *21*, 133–146. https://doi.org/10.1038/s41579-022-00846-2.
22. Meyerholz, D.K. (2023). Rigid respiration: fulminant pulmonary fibrosis after COVID-19. EBioMedicine *87*, 104428. https://doi.org/10.1016/j.ebiom.2022.104428.
23. Hama Amin, B.J., Kakamad, F.H., Ahmed, G.S., Ahmed, S.F., Abdulla, B.A., Mohammed, S.H., Mikael, T.M., Salih, R.Q., Ali, R.K., Salh, A.M., and Hussein, D.A. (2022). Post COVID-19 pulmonary fibrosis; a meta-analysis study. Ann. Med. Surg. *77*, 103590. https://doi.org/10.1016/j.amsu.2022.103590.
24. Lassan, S., Tesar, T., Tisonova, J., and Lassanova, M. (2023). Pharmacological approaches to pulmonary fibrosis following COVID-19. Front. Pharmacol. *14*, 1143158. https://doi.org/10.3389/fphar.2023.1143158.
25. Allen, R.J., Stockwell, A., Oldham, J.M., Guillen-Guio, B., Schwartz, D.A., Maher, T.M., Flores, C., Noth, I., Yaspan, B.L., Jenkins, R.G., et al. (2022). Genome-wide association study across five cohorts identifies five novel loci associated with idiopathic pulmonary fibrosis. Thorax *77*, 829–833. https://doi.org/10.1136/thoraxjnl-2021-218577.
26. Hinrichs, A.S., Karolchik, D., Baertsch, R., Barber, G.P., Bejerano, G., Clawson, H., Diekhans, M., Furey, T.S., Harte, R.A., Hsu, F., et al. (2006). The UCSC Genome Browser Database: update 2006. Nucleic Acids Res. *34*, D590–D598. https://doi.org/10.1093/nar/gkj144.
27. Lawrence, M., Gentleman, R., and Carey, V. (2009). rtracklayer: an R package for interfacing with genome browsers. Bioinformatics *25*, 1841–1842. https://doi.org/10.1093/bioinformatics/btp328.
28. GTEx Consortium (2013). The Genotype-Tissue Expression (GTEx) project. Nat. Genet. *45*, 580–585. https://doi.org/10.1038/ng.2653.
29. Borie, R., Cardwell, J., Konigsberg, I.R., Moore, C.M., Zhang, W., Sasse, S.K., Gally, F., Dobrinskikh, E., Walts, A., Powers, J., et al. (2022). Colocalization of gene expression and DNA methylation with genetic risk variants supports functional roles of MUC5B and DSP in idiopathic pulmonary fibrosis. Am. J. Respir. Crit. Care Med. *206*, 1259–1270. https://doi.org/10.1164/rccm.202110-2308OC.
30. Zhou, W., Triche, T.J., Jr., Laird, P.W., and Shen, H. (2018). SeSAMe: reducing artifactual detection of DNA methylation by Infinium BeadChips in genomic deletions. Nucleic Acids Res. *46*, e123. https://doi.org/10.1093/nar/gky691.
31. Li, M.X., Yeung, J.M.Y., Cherny, S.S., and Sham, P.C. (2012). Evaluating the effective numbers of independent tests and significant p-value thresholds in commercial genotyping arrays and public imputation reference datasets. Hum. Genet. *131*, 747–756. https://doi.org/10.1007/s00439-011-1118-2.
32. Campoy, E., Puig, M., Yakymenko, I., Lerga-Jaso, J., and Cáceres, M. (2022). Genomic architecture and functional effects of potential human inversion supergenes. Philos. Trans. R. Soc. Lond. B Biol. Sci. *377*, 20210209. https://doi.org/10.1098/rstb.2021.0209.
33. Guo, H., Fortune, M.D., Burren, O.S., Schofield, E., Todd, J.A., and Wallace, C. (2015). Integration of disease association and eQTL data using a Bayesian colocalisation approach highlights six candidate causal genes in immune-mediated diseases. Hum. Mol. Genet. *24*, 3305–3313. https://doi.org/10.1093/hmg/ddv077.
34. Jaffar, J., Wong, M., Fishbein, G.A., Alhamdoosh, M., McMillan, L., Gamell-Fulla, C., Ng, M., Wilson, N., Symons, K., Glaspole, I., and Westall, G. (2022). Matrix metalloproteinase-7 is increased in lung bases but not apices in idiopathic pulmonary fibrosis. ERJ Open Res. *8*, 00191-2022. https://doi.org/10.1183/23120541.00191-2022.
35. Sivakumar, P., Thompson, J.R., Ammar, R., Porteous, M., McCoubrey, C., Cantu, E., 3rd, Ravi, K., Zhang, Y., Luo, Y., Streltsov, D., et al. (2019). RNA sequencing of transplant-stage idiopathic pulmonary fibrosis lung reveals unique pathway regulation. ERJ Open Res. *5*, 00117-2019. https://doi.org/10.1183/23120541.00117-2019.
36. Carapito, R., Li, R., Helms, J., Carapito, C., Gujja, S., Rolli, V., Guimaraes, R., Malagon-Lopez, J., Spinnhirny, P., Lederle, A., et al. (2022). Identification of driver genes for critical forms

of COVID-19 in a deeply phenotyped young patient cohort. Sci. Transl. Med. *14*, eabj7521. https://doi.org/10.1126/scitranslmed.abj7521.
37. de Rooij, L.P.M.H., Becker, L.M., Teuwen, L.A., Boeckx, B., Jansen, S., Feys, S., Verleden, S., Liesenborghs, L., Stalder, A.K., Libbrecht, S., et al. (2023). The pulmonary vasculature in lethal COVID-19 and idiopathic pulmonary fibrosis at single-cell resolution. Cardiovasc. Res. *119*, 520–535. https://doi.org/10.1093/cvr/cvac139.
38. Love, M.I., Huber, W., and Anders, S. (2014). Moderated estimation of fold change and dispersion for RNA-seq data with DESeq2. Genome Biol. *15*, 550. https://doi.org/10.1186/s13059-014-0550-8.
39. Adams, T.S., Schupp, J.C., Poli, S., Ayaub, E.A., Neumark, N., Ahangari, F., Chu, S.G., Raby, B.A., DeIuliis, G., Januszyk, M., et al. (2020). Single-cell RNA-seq reveals ectopic and aberrant lung-resident cell populations in idiopathic pulmonary fibrosis. Sci. Adv. *6*, eaba1983. https://doi.org/10.1126/sciadv.aba1983.
40. Melms, J.C., Biermann, J., Huang, H., Wang, Y., Nair, A., Tagore, S., Katsyv, I., Rendeiro, A.F., Amin, A.D., Schapiro, D., et al. (2021). A molecular single-cell lung atlas of lethal COVID-19. Nature *595*, 114–119. https://doi.org/10.1038/s41586-021-03569-1.
41. Tarhan, L., Bistline, J., Chang, J., Galloway, B., Hanna, E., and Weitz, E. (2023). Single Cell Portal: an interactive home for single-cell genomics data. Preprint at bioRxiv. https://doi.org/10.1101/2023.07.13.548886.
42. Wolf, F.A., Angerer, P., and Theis, F.J. (2018). SCANPY: large-scale single-cell gene expression data analysis. Genome Biol. *19*, 15. https://doi.org/10.1186/s13059-017-1382-0.
43. Badia-I-Mompel, P., Vélez Santiago, J., Braunger, J., Geiss, C., Dimitrov, D., Müller-Dott, S., Taus, P., Dugourd, A., Holland, C.H., Ramirez Flores, R.O., and Saez-Rodriguez, J. (2022). decoupleR: ensemble of computational methods to infer biological activities from omics data. Bioinform. Adv. *2*, vbac016. https://doi.org/10.1093/bioadv/vbac016.
44. Muzellec, B., Teleńczuk, M., Cabeli, V., and Andreux, M. (2023). PyDESeq2: a python package for bulk RNA-seq differential expression analysis. Bioinformatics *39*, btad547. https://doi.org/10.1093/bioinformatics/btad547.
45. Valero-Mora, P.M. (2010). ggplot2: elegant graphics for data analysis. J. Stat. Software *35*, 1–3. https://doi.org/10.18637/jss.v035.b01.
46. Paria, S.S., Rahman, S.R., and Adhikari, K. (2022). fastman: A fast algorithm for visualizing GWAS results using Manhattan and Q-Q plots. Preprint at bioRxiv. https://doi.org/10.1101/2022.04.19.488738.
47. COVID-19 Host Genetics Initiative (2022). A first update on mapping the human genetic architecture of COVID-19. Nature *608*. E1-e10. https://doi.org/10.1038/s41586-022-04826-7.
48. Writing group Writing group leader (2021). Mapping the human genetic architecture of COVID-19. Nature *600*, 472–477. https://doi.org/10.1038/s41586-021-03767-x.
49. Ward, L.D., and Kellis, M. (2012). HaploReg: a resource for exploring chromatin states, conservation, and regulatory motif alterations within sets of genetically linked variants. Nucleic Acids Res. *40*, D930–D934. https://doi.org/10.1093/nar/gkr917.
50. Helling, B.A., Gerber, A.N., Kadiyala, V., Sasse, S.K., Pedersen, B.S., Sparks, L., Nakano, Y., Okamoto, T., Evans, C.M., Yang, I.V., and Schwartz, D.A. (2017). Regulation of MUC5B Expression in Idiopathic Pulmonary Fibrosis. Am. J. Respir. Cell Mol. Biol. *57*, 91–99. https://doi.org/10.1165/rcmb.2017-0046OC.
51. Connally, N.J., Nazeen, S., Lee, D., Shi, H., Stamatoyannopoulos, J., Chun, S., Cotsapas, C., Cassa, C.A., and Sunyaev, S.R. (2022). The missing link between genetic association and regulatory function. Elife *11*, e74970. https://doi.org/10.7554/eLife.74970.
52. Willett, J.D.S., Lu, T., Nakanishi, T., Yoshiji, S., Butler-Laporte, G., Zhou, S., Farjoun, Y., and Richards, J.B. (2023). Colocalization of expression transcripts with COVID-19 outcomes is rare across cell states, cell types and organs. Hum. Genet. *142*, 1461–1476. https://doi.org/10.1007/s00439-023-02590-w.
53. Hao, K., Bossé, Y., Nickle, D.C., Paré, P.D., Postma, D.S., Laviolette, M., Sandford, A., Hackett, T.L., Daley, D., Hogg, J.C., et al. (2012). Lung eQTLs to help reveal the molecular underpinnings of asthma. PLoS Genet. *8*, e1003029. https://doi.org/10.1371/journal.pgen.1003029.
54. Zhu, J., Zhou, D., Yu, M., and Li, Y. (2024). Appraising the causal role of smoking in idiopathic pulmonary fibrosis: a Mendelian randomization study. Thorax *79*, 179–181. https://doi.org/10.1136/thorax-2023-220012.
55. Margaritopoulos, G.A., Vasarmidi, E., Jacob, J., Wells, A.U., and Antoniou, K.M. (2015). Smoking and interstitial lung diseases. Eur. Respir. Rev. *24*, 428–435. https://doi.org/10.1183/16000617.0050-2015.
56. Baumgartner, K.B., Samet, J.M., Stidley, C.A., Colby, T.V., and Waldron, J.A. (1997). Cigarette smoking: a risk factor for idiopathic pulmonary fibrosis. Am. J. Respir. Crit. Care Med. *155*, 242–248. https://doi.org/10.1164/ajrccm.155.1.9001319.
57. Elisia, I., Lam, V., Cho, B., Hay, M., Li, M.Y., Yeung, M., Bu, L., Jia, W., Norton, N., Lam, S., and Krystal, G. (2020). The effect of smoking on chronic inflammation, immune function and blood cell composition. Sci. Rep. *10*, 19480. https://doi.org/10.1038/s41598-020-76556-7.
58. Lugg, S.T., Scott, A., Parekh, D., Naidu, B., and Thickett, D.R. (2022). Cigarette smoke exposure and alveolar macrophages: mechanisms for lung disease. Thorax *77*, 94–101. https://doi.org/10.1136/thoraxjnl-2020-216296.
59. Wohnhaas, C.T., Baßler, K., Watson, C.K., Shen, Y., Leparc, G.G., Tilp, C., Heinemann, F., Kind, D., Stierstorfer, B., Delić, D., et al. (2024). Monocyte-derived alveolar macrophages are key drivers of smoke-induced lung inflammation and tissue remodeling. Front. Immunol. *15*, 1325090. https://doi.org/10.3389/fimmu.2024.1325090.
60. Chen, L., Ge, B., Casale, F.P., Vasquez, L., Kwan, T., Garrido-Martín, D., Watt, S., Yan, Y., Kundu, K., Ecker, S., et al. (2016). Genetic Drivers of Epigenetic and Transcriptional Variation in Human Immune Cells. Cell *167*, 1398–1414.e24. https://doi.org/10.1016/j.cell.2016.10.026.
61. Ota, M., Nagafuchi, Y., Hatano, H., Ishigaki, K., Terao, C., Takeshima, Y., Yanaoka, H., Kobayashi, S., Okubo, M., Shirai, H., et al. (2021). Dynamic landscape of immune cell-specific gene regulation in immune-mediated diseases. Cell *184*, 3006–3021.e17. https://doi.org/10.1016/j.cell.2021.03.056.
62. Kapellos, T.S., Bonaguro, L., Gemünd, I., Reusch, N., Saglam, A., Hinkley, E.R., and Schultze, J.L. (2019). Human Monocyte Subsets and Phenotypes in Major Chronic Inflammatory Diseases. Front. Immunol. *10*, 2035. https://doi.org/10.3389/fimmu.2019.02035.

63. Willis, B.C., duBois, R.M., and Borok, Z. (2006). Epithelial origin of myofibroblasts during fibrosis in the lung. Proc. Am. Thorac. Soc. *3*, 377–382. https://doi.org/10.1513/pats.200601-004TK.
64. Phan, S.H. (2012). Genesis of the myofibroblast in lung injury and fibrosis. Proc. Am. Thorac. Soc. *9*, 148–152. https://doi.org/10.1513/pats.201201-011AW.
65. Lin, Y., and Xu, Z. (2020). Fibroblast Senescence in Idiopathic Pulmonary Fibrosis. Front. Cell Dev. Biol. *8*, 593283. https://doi.org/10.3389/fcell.2020.593283.
66. Peyser, R., MacDonnell, S., Gao, Y., Cheng, L., Kim, Y., Kaplan, T., Ruan, Q., Wei, Y., Ni, M., Adler, C., et al. (2019). Defining the Activated Fibroblast Population in Lung Fibrosis Using Single-Cell Sequencing. Am. J. Respir. Cell Mol. Biol. *61*, 74–85. https://doi.org/10.1165/rcmb.2018-0313OC.
67. Aquino, Y., Bisiaux, A., Li, Z., O'Neill, M., Mendoza-Revilla, J., Merkling, S.H., Kerner, G., Hasan, M., Libri, V., Bondet, V., et al. (2023). Dissecting human population variation in single-cell responses to SARS-CoV-2. Nature *621*, 120–128. https://doi.org/10.1038/s41586-023-06422-9.
68. Natri, H.M., Del Azodi, C.B., Peter, L., Taylor, C.J., Chugh, S., Kendle, R., Chung, M.I., Flaherty, D.K., Matlock, B.K., Calvi, C.L., et al. (2024). Cell-type-specific and disease-associated expression quantitative trait loci in the human lung. Nat. Genet. *56*, 595–604. https://doi.org/10.1038/s41588-024-01702-0.
69. Gaunt, T.R., Shihab, H.A., Hemani, G., Min, J.L., Woodward, G., Lyttleton, O., Zheng, J., Duggirala, A., McArdle, W.L., Ho, K., et al. (2016). Systematic identification of genetic influences on methylation across the human life course. Genome Biol. *17*, 61. https://doi.org/10.1186/s13059-016-0926-z.
70. McRae, A.F., Marioni, R.E., Shah, S., Yang, J., Powell, J.E., Harris, S.E., Gibson, J., Henders, A.K., Bowdler, L., Painter, J.N., et al. (2018). Identification of 55,000 Replicated DNA Methylation QTL. Sci. Rep. *8*, 17605. https://doi.org/10.1038/s41598-018-35871-w.
71. Moore, L.D., Le, T., and Fan, G. (2013). DNA methylation and its basic function. Neuropsychopharmacology *38*, 23–38. https://doi.org/10.1038/npp.2012.112.
72. Perrot, C.Y., Karampitsakos, T., and Herazo-Maya, J.D. (2023). Monocytes and macrophages: emerging mechanisms and novel therapeutic targets in pulmonary fibrosis. Am. J. Physiol. Cell Physiol. *325*, C1046–C1057. https://doi.org/10.1152/ajpcell.00302.2023.
73. Hancock, L.A., Hennessy, C.E., Solomon, G.M., Dobrinskikh, E., Estrella, A., Hara, N., Hill, D.B., Kissner, W.J., Markovetz, M.R., Grove Villalon, D.E., et al. (2018). Muc5b overexpression causes mucociliary dysfunction and enhances lung fibrosis in mice. Nat. Commun. *9*, 5363. https://doi.org/10.1038/s41467-018-07768-9.
74. Kato, T., Asakura, T., Edwards, C.E., Dang, H., Mikami, Y., Okuda, K., Chen, G., Sun, L., Gilmore, R.C., Hawkins, P., et al. (2022). Prevalence and Mechanisms of Mucus Accumulation in COVID-19 Lung Disease. Am. J. Respir. Crit. Care Med. *206*, 1336–1352. https://doi.org/10.1164/rccm.202111-2606OC.
75. Yan, J., Li, Q., Mao, A.P., Hu, M.M., and Shu, H.B. (2014). TRIM4 modulates type I interferon induction and cellular antiviral response by targeting RIG-I for K63-linked ubiquitination. J. Mol. Cell Biol. *6*, 154–163. https://doi.org/10.1093/jmcb/mju005.
76. Zhang, X., Yang, Z., Pan, T., Sun, Q., Chen, Q., Wang, P.H., Li, X., and Kuang, E. (2023). SARS-CoV-2 Nsp8 suppresses MDA5 antiviral immune responses by impairing TRIM4-mediated K63-linked polyubiquitination. PLoS Pathog. *19*, e1011792. https://doi.org/10.1371/journal.ppat.1011792.
77. Li, C., Wang, Z., Zhang, J., Zhao, X., Xu, P., Liu, X., Li, M., Lv, C., and Song, X. (2019). Crosstalk of mRNA, miRNA, lncRNA, and circRNA and Their Regulatory Pattern in Pulmonary Fibrosis. Mol. Ther. Nucleic Acids *18*, 204–218. https://doi.org/10.1016/j.omtn.2019.08.018.
78. Jiang, X., and Ning, Q. (2019). Circular RNAs as novel regulators, biomarkers and potential therapies in fibrosis. Epigenomics *11*, 1107–1116. https://doi.org/10.2217/epi-2019-0001.
79. Yao, Z., Luo, J., Hu, K., Lin, J., Huang, H., Wang, Q., Zhang, P., Xiong, Z., He, C., Huang, Z., et al. (2017). ZKSCAN1 gene and its related circular RNA (circZKSCAN1) both inhibit hepatocellular carcinoma cell growth, migration, and invasion but through different signaling pathways. Mol. Oncol. *11*, 422–437. https://doi.org/10.1002/1878-0261.12045.
80. Khalil, N., Bereznay, O., Sporn, M., and Greenberg, A.H. (1989). Macrophage production of transforming growth factor beta and fibroblast collagen synthesis in chronic pulmonary inflammation. J. Exp. Med. *170*, 727–737. https://doi.org/10.1084/jem.170.3.727.
81. Takatsu, H., Tanaka, G., Segawa, K., Suzuki, J., Nagata, S., Nakayama, K., and Shin, H.W. (2014). Phospholipid flippase activities and substrate specificities of human type IV P-type ATPases localized to the plasma membrane. J. Biol. Chem. *289*, 33543–33556. https://doi.org/10.1074/jbc.M114.593012.
82. Segawa, K., Kurata, S., and Nagata, S. (2016). Human Type IV P-type ATPases That Work as Plasma Membrane Phospholipid Flippases and Their Regulation by Caspase and Calcium. J. Biol. Chem. *291*, 762–772. https://doi.org/10.1074/jbc.M115.690727.
83. Nagata, S., Suzuki, J., Segawa, K., and Fujii, T. (2016). Exposure of phosphatidylserine on the cell surface. Cell Death Differ. *23*, 952–961. https://doi.org/10.1038/cdd.2016.7.
84. Segawa, K., and Nagata, S. (2015). An Apoptotic 'Eat Me' Signal: Phosphatidylserine Exposure. Trends Cell Biol. *25*, 639–650. https://doi.org/10.1016/j.tcb.2015.08.003.
85. Drakopanagiotakis, F., Xifteri, A., Polychronopoulos, V., and Bouros, D. (2008). Apoptosis in lung injury and fibrosis. Eur. Respir. J. *32*, 1631–1638. https://doi.org/10.1183/09031936.00176807.
86. Uhal, B.D. (2008). The role of apoptosis in pulmonary fibrosis. Eur. Respir. Rev. *17*, 138–144. https://doi.org/10.1183/09059180.00010906.
87. Kim, K.K., Dotson, M.R., Agarwal, M., Yang, J., Bradley, P.B., Subbotina, N., Osterholzer, J.J., and Sisson, T.H. (2018). Efferocytosis of apoptotic alveolar epithelial cells is sufficient to initiate lung fibrosis. Cell Death Dis. *9*, 1056. https://doi.org/10.1038/s41419-018-1074-z.
88. Morimoto, K., Janssen, W.J., and Terada, M. (2012). Defective efferocytosis by alveolar macrophages in IPF patients. Respir. Med. *106*, 1800–1803. https://doi.org/10.1016/j.rmed.2012.08.020.
89. van der Mark, V.A., Ghiboub, M., Marsman, C., Zhao, J., van Dijk, R., Hiralall, J.K., Ho-Mok, K.S., Castricum, Z., de Jonge, W.J., Oude Elferink, R.P.J., and Paulusma, C.C. (2017). Phospholipid flippases attenuate LPS-induced TLR4 signaling by

mediating endocytic retrieval of Toll-like receptor 4. Cell. Mol. Life Sci. *74*, 715–730. https://doi.org/10.1007/s00018-016-2360-5.

90. Liang, J., Zhang, Y., Xie, T., Liu, N., Chen, H., Geng, Y., Kurkciyan, A., Mena, J.M., Stripp, B.R., Jiang, D., and Noble, P.W. (2016). Hyaluronan and TLR4 promote surfactant-protein-C-positive alveolar progenitor cell renewal and prevent severe pulmonary fibrosis in mice. Nat. Med. *22*, 1285–1293. https://doi.org/10.1038/nm.4192.
91. Sahanic, S., Hilbe, R., Dünser, C., Tymoszuk, P., Löffler-Ragg, J., Rieder, D., Trajanoski, Z., Krogsdam, A., Demetz, E., Yurchenko, M., et al. (2023). SARS-CoV-2 activates the TLR4/MyD88 pathway in human macrophages: A possible correlation with strong pro-inflammatory responses in severe COVID-19. Heliyon *9*, e21893. https://doi.org/10.1016/j.heliyon.2023.e21893.
92. Zhao, Y., Kuang, M., Li, J., Zhu, L., Jia, Z., Guo, X., Hu, Y., Kong, J., Yin, H., Wang, X., and You, F. (2021). SARS-CoV-2 spike protein interacts with and activates TLR41. Cell Res. *31*, 818–820. https://doi.org/10.1038/s41422-021-00495-9.
93. Ouyang, W., Xie, T., Fang, H., Gao, C., Stantchev, T., Clouse, K.A., Yuan, K., Ju, T., and Frucht, D.M. (2021). Variable Induction of Pro-Inflammatory Cytokines by Commercial SARS CoV-2 Spike Protein Reagents: Potential Impacts of LPS on In Vitro Modeling and Pathogenic Mechanisms In Vivo. Int. J. Mol. Sci. *22*, 7540. https://doi.org/10.3390/ijms22147540.
94. Cinquegrani, G., Spigoni, V., Iannozzi, N.T., Parello, V., Bonadonna, R.C., and Dei Cas, A. (2022). SARS-CoV-2 Spike protein is not pro-inflammatory in human primary macrophages: endotoxin contamination and lack of protein glycosylation as possible confounders. Cell Biol. Toxicol. *38*, 667–678. https://doi.org/10.1007/s10565-021-09693-y.
95. Petruk, G., Puthia, M., Petrlova, J., Samsudin, F., Strömdahl, A.C., Cerps, S., Uller, L., Kjellström, S., Bond, P.J., and Schmidtchen, A.A. (2020). SARS-CoV-2 spike protein binds to bacterial lipopolysaccharide and boosts proinflammatory activity. J. Mol. Cell Biol. *12*, 916–932. https://doi.org/10.1093/jmcb/mjaa067.
96. Samsudin, F., Raghuvamsi, P., Petruk, G., Puthia, M., Petrlova, J., MacAry, P., Anand, G.S., Bond, P.J., and Schmidtchen, A. (2023). SARS-CoV-2 spike protein as a bacterial lipopolysaccharide delivery system in an overzealous inflammatory cascade. J. Mol. Cell Biol. *14*, mjac058. https://doi.org/10.1093/jmcb/mjac058.
97. Hollingsworth, L.R., Sharif, H., Griswold, A.R., Fontana, P., Mintseris, J., Dagbay, K.B., Paulo, J.A., Gygi, S.P., Bachovchin, D.A., and Wu, H. (2021). DPP9 sequesters the C terminus of NLRP1 to repress inflammasome activation. Nature *592*, 778–783. https://doi.org/10.1038/s41586-021-03350-4.
98. Okondo, M.C., Rao, S.D., Taabazuing, C.Y., Chui, A.J., Poplawski, S.E., Johnson, D.C., and Bachovchin, D.A. (2018). Inhibition of Dpp8/9 Activates the Nlrp1b Inflammasome. Cell Chem. Biol. *25*, 262–267.e5. https://doi.org/10.1016/j.chembiol.2017.12.013.
99. Zhong, F.L., Robinson, K., Teo, D.E.T., Tan, K.Y., Lim, C., Harapas, C.R., Yu, C.H., Xie, W.H., Sobota, R.M., Au, V.B., et al. (2018). Human DPP9 represses NLRP1 inflammasome and protects against autoinflammatory diseases via both peptidase activity and FIIND domain binding. J. Biol. Chem. *293*, 18864–18878. https://doi.org/10.1074/jbc.RA118.004350.
100. Gabrilovac, J., Čupić, B., Zapletal, E., Kraus, O., and Jakić-Razumović, J. (2017). Dipeptidyl peptidase 9 (DPP9) in human skin cells. Immunobiology *222*, 327–342. https://doi.org/10.1016/j.imbio.2016.09.007.
101. Acharya, P.S., Zukas, A., Chandan, V., Katzenstein, A.L.A., and Puré, E. (2006). Fibroblast activation protein: a serine protease expressed at the remodeling interface in idiopathic pulmonary fibrosis. Hum. Pathol. *37*, 352–360. https://doi.org/10.1016/j.humpath.2005.11.020.
102. Wu, Q.Q., Zhao, M., Huang, G.Z., Zheng, Z.N., Chen, Y., Zeng, W.S., and Lv, X.Z. (2020). Fibroblast Activation Protein (FAP) Overexpression Induces Epithelial-Mesenchymal Transition (EMT) in Oral Squamous Cell Carcinoma by Down-Regulating Dipeptidyl Peptidase 9 (DPP9). OncoTargets Ther. *13*, 2599–2611. https://doi.org/10.2147/ott.S243417.
103. Nakanishi, T., Willett, J., Farjoun, Y., Allen, R.J., Guillen-Guio, B., Adra, D., Zhou, S., and Richards, J.B. (2023). Alternative splicing in lung influences COVID-19 severity and respiratory diseases. Nat. Commun. *14*, 6198. https://doi.org/10.1038/s41467-023-41912-4.
104. Mahida, R., and Turner, A.M. (2013). MUC5B: a good target for future therapy in pulmonary fibrosis? Thorax *68*, 401. https://doi.org/10.1136/thoraxjnl-2012-202957.
105. Benramdane, S., De Loose, J., Filippi, N., Espadinha, M., Beyens, O., Rymenant, Y.V., Dirkx, L., Bozdag, M., Feijens, P.B., Augustyns, K., et al. (2023). Highly Selective Inhibitors of Dipeptidyl Peptidase 9 (DPP9) Derived from the Clinically Used DPP4-Inhibitor Vildagliptin. J. Med. Chem. *66*, 12717–12738. https://doi.org/10.1021/acs.jmedchem.3c00609.

HGGA, Volume *6*

Supplemental information

Context-specific eQTLs provide deeper insight into causal genes underlying shared genetic architecture of COVID-19 and idiopathic pulmonary fibrosis

Trisha Dalapati, Liuyang Wang, Angela G. Jones, Jonathan Cardwell, Iain R. Konigsberg, Yohan Bossé, Don D. Sin, Wim Timens, Ke Hao, Ivana Yang, and Dennis C. Ko

A

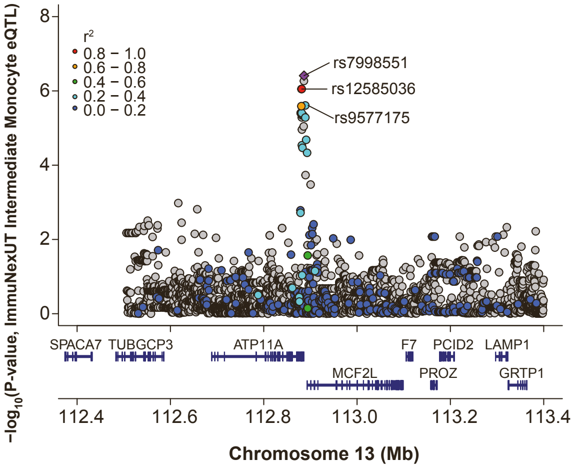

B

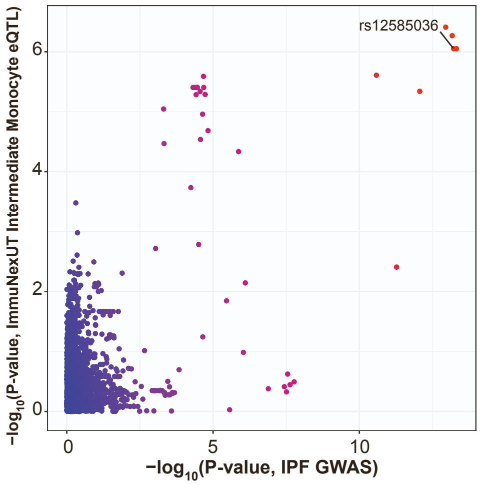

C

COLOC of the ImmuNexUT Intermediate Monocyte eQTL and Disease GWAS Signals at *ATP11A*

rsID	Disease	PP3	PP4	PP4/PP3	Interpretation
**rs12585036**	IPF	9.83×10^{-3}	0.988	1.01×10^{2}	Traits share a single causal variant.
	COVID-19	8.88×10^{-3}	0.989	1.11×10^{2}	Traits share a single causal variant.

Figure S1: Colocalization at rs12585036 reveals *ATP11A* as a causal gene in monocytes from a Japanese cohort.

(A) rs12585036 is a top eQTL and in strong LD with the lead eQTL (rs7998551) for *ATP11A* in monocytes, most significantly in intermediate monocytes, in the ImmuNexUT eQTL atlas.

(B) Comparison of -$\log_{10}$(p-values) from the IPF GWAS and intermediate monocytic *ATP11A*-eQTLs shows rs12585036 as a top shared SNP in ImmuNexUT.

(C) COLOC indicates strong colocalization between the disease GWAS and monocytic *ATP11A*-eQTLs signals in ImmuNexUT with PP4 > 0.900 and PP4/PP3 > 5.00

P-values are -$\log_{10}$ transformed. Linkage disequilibrium information for the Japanese population was obtained from LDlink and is relative to the lead variant in each plot.

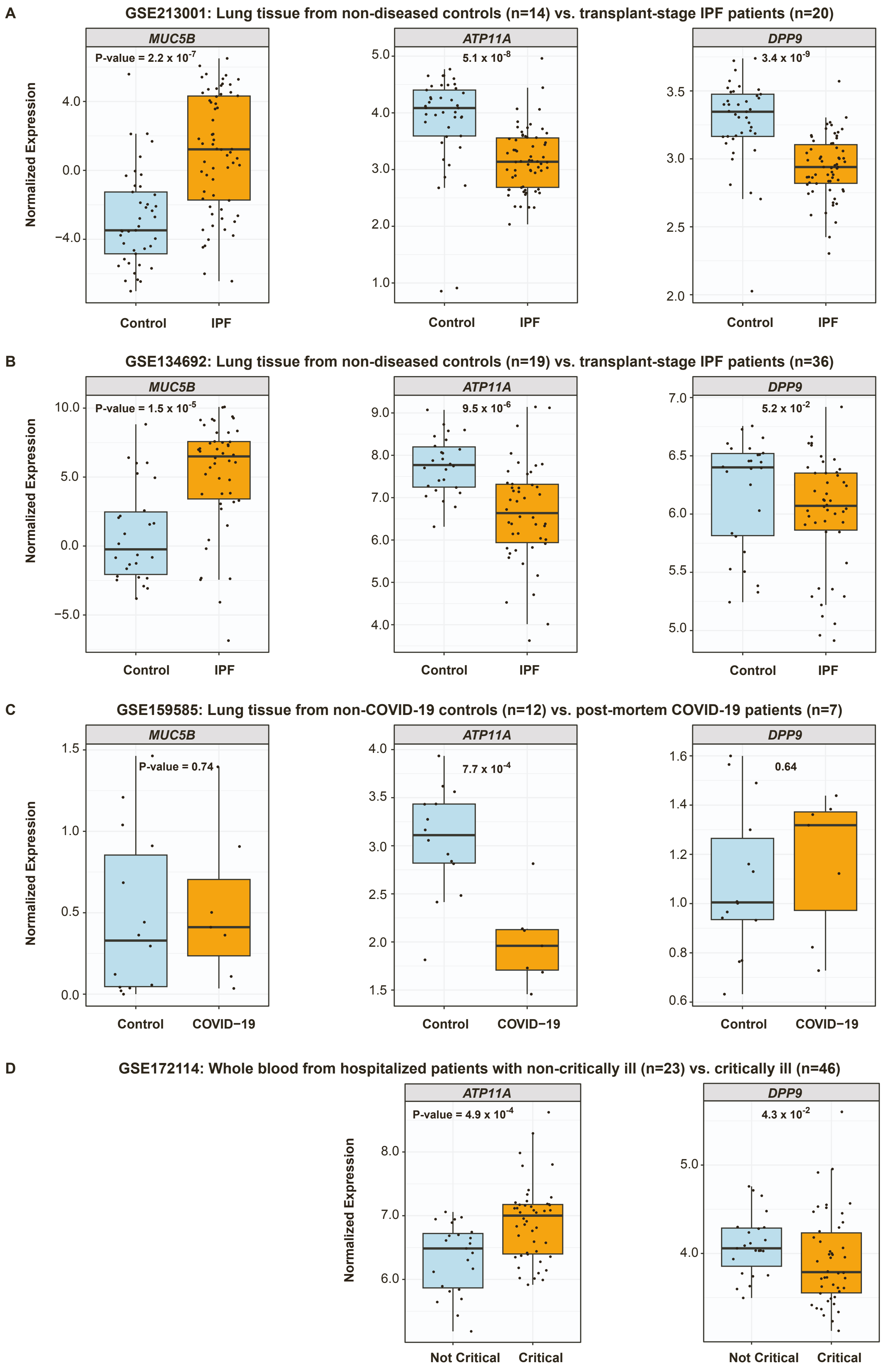
A
GSE213001: Lung tissue from non-diseased controls (n=14) vs. transplant-stage IPF patients (n=20)
MUC5B
P-value = 2.2 x 10-7
ATP11A
5.1 x 10-8
DPP9
3.4 x 10-9
Normalized Expression
Control
IPF
B
GSE134692: Lung tissue from non-diseased controls (n=19) vs. transplant-stage IPF patients (n=36)
MUC5B
P-value = 1.5 x 10-5
ATP11A
9.5 x 10-6
DPP9
5.2 x 10-2
Normalized Expression
Control
IPF
C
GSE159585: Lung tissue from non-COVID-19 controls (n=12) vs. post-mortem COVID-19 patients (n=7)
MUC5B
P-value = 0.74
ATP11A
7.7 x 10-4
DPP9
0.64
Normalized Expression
Control
COVID−19
D
GSE172114: Whole blood from hospitalized patients with non-critically ill (n=23) vs. critically ill (n=46)
ATP11A
P-value = 4.9 x 10-4
DPP9
4.3 x 10-2
Normalized Expression
Not Critical
Critical

Figure S2: Assessment of causal genes in bulk control and diseased tissue from independent studies.

Boxplots depict normalized expression of causal genes by diagnosis from:

(A) GSE213001, which included lung tissue from non-diseased controls (n=14) and IPF patients undergoing lung transplant (n=20).

(B) GSE134692, which included lung tissue from non-diseased controls (n=19) and IPF patients undergoing lung transplant (n=36).

(C) GSE159585, which included lung tissue from non-diseased controls (n=12) and from patients who died from COVID-19 (n=7).

(D) GSE172114, which included whole blood samples from hospitalized patients with non-critically ill COVID-19 (n=23) and patients in the intensive care unit with critically ill COVID-19 (n=46).

P-values were calculated using the Mann-Whitney U test.

A

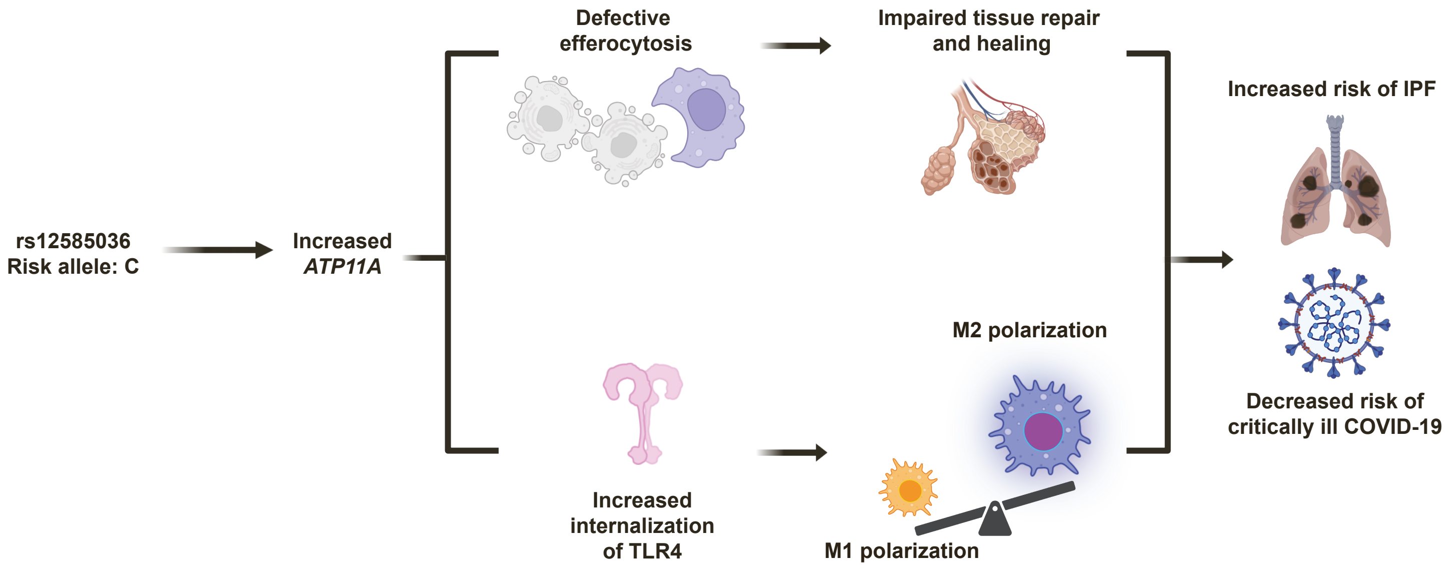
rs12585036
Risk allele: C
Increased
ATP11A
Defective
efferocytosis
Impaired tissue repair
and healing
Increased
internalization
of TLR4
M2 polarization
M1 polarization
Increased risk of IPF
Decreased risk of
critically ill COVID-19

B

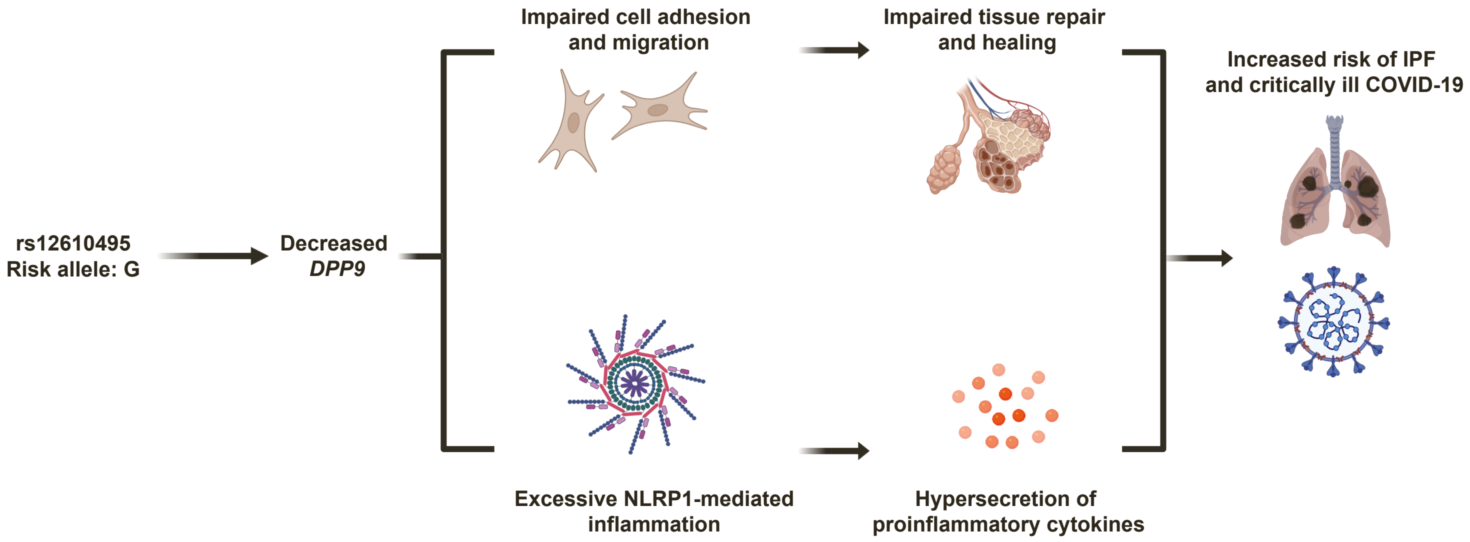
rs12610495
Risk allele: G
Decreased
DPP9
Impaired cell adhesion
and migration
Impaired tissue repair
and healing
Excessive NLRP1-mediated
inflammation
Hypersecretion of
proinflammatory cytokines
Increased risk of IPF
and critically ill COVID-19

Figure S3: Hypothesized mechanisms linking disease-associated SNPs to causal genes, *ATP11A* and *DPP9*.

(A) The C allele of rs12585036 is associated with increased *ATP11A*, leading to 1) defective efferocytosis and impaired lung tissue repair and 2) increased internalization of toll-like receptor 4 and increased M2-macrophage polarization. These mechanisms lead to increased risk of IPF and protection from critically ill COVID-19.

(B) The G allele of rs12610495 is associated with decreased *DPP9*, leading to 1) impaired cell adhesion and migration affecting lung tissue repair, and 2) excessive NLRP1-inflammasome activity and proinflammatory cytokine production. These mechanisms lead to increased risk of both IPF and critically ill COVID-19.
